# Biobased Polymer Composites for Sensor-Integrated Wound Dressings: Structural Roles, Sensing Interfaces and Validation Challenges

**DOI:** 10.3390/polym18212607

**Published:** 2026-10-22

**Authors:** Jia-Ling Zhu, Yuexiang Yang, Yunfei Mo, Chih-Hao Chen, Eric Hald, Chia-Lin Sheu

**Affiliations:** 1 Department of Dermatology, Dongguan Hospital of Guangzhou University of Chinese Medicine, Dongguan 523000, China; jolinjialingzhu@hotmail.com; 2 School of Mechanical Engineering, Dongguan University of Technology, Dongguan 523808, China; 2024411120128@dgut.edu.cn; 3 Department of Biomedical Engineering, Dongguan University of Technology, Dongguan 523808, China; mofaaron227@dgut.edu.cn; 4 Department of Plastic and Reconstructive Surgery, Chang Gung Memorial Hospital, Keelung & Chang Gung University, Taoyuan 33302, Taiwan; 5 Department of Biomedical Engineering, University of Michigan, Ann Arbor, MI 48109, USA; ehald@umich.edu

**Keywords:** biobased polymer composites, sensor-integrated wound dressings, smart wound dressings, wound exudate monitoring, hydrogel dressings, conductive biomaterials, colorimetric sensing, electrochemical biosensors, interface stability, validation

## Abstract

Sensor-integrated wound dressings place optical, electrochemical, biochemical, or wireless sensing modules within materials that are simultaneously responsible for wound contact and fluid management. In this setting, biobased polymers influence hydration, swelling, capillary transport, wet-state mechanics, retention of functional phases, and the local environment presented to the sensor. This review examines how chitosan, alginate, hyaluronic acid, cellulose, nanocellulose, bacterial cellulose, gelatin, collagen, and silk fibroin function as wound-contact layers, absorbent reservoirs, capillary supports, transition domains, and hosts for sensing components. Auxiliary phases, including PVA, PEDOT:PSS, carbon nanomaterials, MXenes, metal nanoparticles, responsive dyes, enzymes, and antibody-based recognition elements, are considered according to the conductivity, optical response, catalytic activity, antimicrobial function, or molecular selectivity they introduce into these matrices. Across the reviewed studies, signal reliability varied with exudate handling, sensing-zone exposure, fouling, wet-state stability, and the spatial organization of functional modules. We use these relationships to examine how material architecture stabilizes the sensing interface and to interpret validation in the wound-monitoring conditions under which each readout was tested.

## 1. Introduction

Hard-to-heal wounds remain a major clinical and socioeconomic burden because healing occurs within a changing local environment shaped by inflammation, tissue remodeling, microbial burden, exudate production, and persistent impairment of repair [1,2]. Modern dressings therefore do more than provide protection and absorb fluid; they also regulate moisture and increasingly serve as platforms for local therapy and monitoring [3,4]. In sensor-integrated systems, the analytical signal is generated at a wet, biologically complex interface. Measurements of pH, temperature, oxygenation, microbial activity, protease activity, and inflammatory mediators are consequently influenced by the wound fluid in which these signals are sampled. Reviews of wound-exudate biomarkers identify these analytes as potential sources of wound-state information, whereas studies of wearable body-fluid analysis and biosensor matrix effects demonstrate that sampling media and biological background can substantially affect analytical reliability [5,6].

Recent smart wound platforms have extended monitoring to several of these physicochemical and infection-related signals. Stretchable, wireless, and theranostic configurations further combine sensing with antibacterial or anti-inflammatory treatment, drug release, or electrical stimulation in soft wound-contact devices [7,8]. These functions make the surrounding material architecture part of the analytical system because the dressing determines how wound fluid is contacted, transported, retained, and presented to the sensing region. The measured response is consequently determined by both the sensing chemistry and the way the dressing absorbs, renews, or stores exudate, together with interference from proteins, cells, bacteria, and other wound-fluid components [7].

Reported wound-fluid values should be treated as clinical ranges instead of universal diagnostic thresholds. Healthy skin and uncomplicated healing wounds are generally acidic (approximately pH 4–6 or 5–6), whereas chronic or infected wound fluid is commonly reported at pH 7–9; chronic-wound measurements of pH 7.15–8.9 and values up to 9.25 have also been described [4,8,9]. Exudate output is likewise method- and wound-size-dependent: dressing-weight studies of leg ulcers reported approximately 0.10–0.21 g cm−2 per 24 h, while pragmatic categories classify less than 5, 5–10, and more than 10 mL per 24 h as low, moderate, and high exudation, respectively [10]. Venous leg ulcers are often persistently exudative because of venous hypertension and edema; diabetic foot ulcers range from relatively dry ischemic lesions to highly exudative infected wounds; and burns can show substantial early fluid loss and broad chemical variation. Sensor range, fluid-handling capacity, and sampling interval should be matched to wound etiology, size, healing stage, infection status, and dressing-change interval instead of to a single nominal wound-fluid condition [3,7,9,10].

Biobased polymer composites are relevant to these problems because natural matrices can provide wound-compatible contact, hydration, capillary action, reservoir behavior, soft mechanical transition, and functional-phase hosting. Chitosan, alginate, hyaluronic acid (HA), cellulose, nanocellulose, bacterial cellulose, gelatin, collagen, and silk fibroin are commonly used as wound-contact or hydrated support domains [11,12]. Auxiliary components such as poly(vinyl alcohol) (PVA), poly(3,4-ethylenedioxythiophene):poly(styrenesulfonate) (PEDOT:PSS), MXenes, metal nanoparticles, carbon nanomaterials, responsive dyes, enzymes, and other recognition elements provide mechanical reinforcement, conductivity, antibacterial activity, optical response, or biochemical selectivity [13,14]. Broad biocompatibility alone does not define suitability for sensing; molecular and network variables also determine how a sample is transported and presented to the sensing interface.

For this review, a biobased composite is operationally defined as a dressing architecture in which at least one material domain that directly provides wound contact, exudate handling, structural support, or hosting of sensing chemistry is derived wholly or partly from renewable biological feedstock. No minimum mass fraction is imposed because many device reports do not disclose component fractions and because a thin biobased contact or routing layer can govern sample presentation despite contributing little bulk mass. Synthetic polymers, conductive phases, microfluidics, and electronics are included when they reinforce or interrogate a functionally relevant biobased domain; systems in which a biobased ingredient is merely incidental and does not influence the wound-contact, transport, structural, or sensing role are excluded.

Existing reviews provide complementary but largely separate perspectives. Broad wound-technology reviews emphasize clinical functions and translation [3], smart-dressing reviews organize systems by monitored biological parameter or by sensing and therapeutic modality [4,8], wearable-fluid reviews focus on sensing modalities and body-fluid analysis [5], and natural-polymer or conductive-biomaterial reviews organize evidence by matrix family or therapeutic function [11,12,13,14]. These syntheses do not consistently connect polymer molecular characteristics and network architecture with exudate transport, sensing-zone exposure, wet-state drift, and the strength of validation supporting each readout. The present review addresses that gap through a material architecture-sample pathway-sensing interface-validation framework. Its analytical contribution is to trace how a wound-derived input is modified before signal generation and to identify the evidence needed to distinguish material responsiveness from reliable wound monitoring.

The distinction in Table 1 is the unit of analysis, not a claim that earlier reviews omit materials, sensing, or translation. Broad and scoping reviews establish the clinical and technological landscape, whereas the present review asks a narrower causal question: how does a biobased polymer composite alter the sample that eventually reaches the sensing interface, and what evidence is required before that response can be interpreted as wound monitoring? This question requires polymer variables, fluid transport, interface stability, and validation strength to be evaluated within the same analytical chain.

We organize the review around the route taken by wound fluid through a sensor-integrated dressing. Polymer matrices, composite domains, exudate-handling structures, sensing interfaces, and wound-contact materials are considered when they alter sampling, signal stability, or readout reliability. Device-level studies are included when their architecture clarifies sensing-zone exposure, interface partitioning, wet-use stability, validation depth, or manufacturable integration. This organization preserves the article’s material focus while providing a reproducible route from polymer design to analytical and translational evidence.

### Literature Search Strategy and Review Scope

This article is a narrative review. Literature searches were conducted in PubMed, Crossref, and OpenAlex and were supplemented by backward and forward citation searching of relevant reviews and primary studies. The final search was completed on 8 October 2026. Searches primarily covered January 2010 to October 2026; earlier studies were retained when they established a material, sensing, or validation principle used by later wound-dressing systems. English-language journal articles and reviews were considered.

The core Boolean structure combined (“wound dressing” OR bandage OR hydrogel) AND (sensor* OR monitor* OR biosensor* OR colorimetric OR electrochemical OR wireless) AND (biopolymer OR biobased OR chitosan OR alginate OR “hyaluronic acid” OR cellulose OR gelatin OR collagen OR silk) AND (exudate OR pH OR temperature OR oxygen OR “uric acid” OR protease OR infection). Secondary searches added swelling, crosslinking, fluid transport, capillary, fouling, sterilization, storage, stability, validation, and clinical terms. Primary studies were included when they described a sensor-integrated or sample-routing wound platform and reported material architecture, analytical performance, fluid exposure, wet-state behavior, or biological validation. Purely therapeutic dressings without a monitoring or sample-pathway contribution were excluded, except where they supplied directly relevant evidence on fluid handling, sterilization, storage, or biosafety. Because device designs, analytes, test media, and outcomes were heterogeneous, no pooled meta-analysis or formal risk-of-bias score was applied; evidence was synthesized by material role, transport pathway, readout mechanism, and validation level.

## 2. Matrix Architecture and Sensing-Interface Design in Biobased Composite Wound Dressings

### 2.1. Biobased Polymer Matrices as Wound-Contact and Sensing-Support Domains

For sensing applications, the function of a biobased polymer within the dressing architecture is more important than its biological origin. Water uptake, swelling kinetics, pore architecture, charge density, degradation behavior, and wet-state mechanics determine whether a matrix functions mainly as a wound-contact layer, an exudate reservoir, a capillary support, a mechanical transition layer, or a host for responsive chemistry [15]. These properties affect the local concentration and residence time of analytes presented to a sensor. Matrix-hosted optical and enzymatic readouts add a parallel requirement: the responsive phase must remain both retained and accessible within the hydrated network.

**Figure 1:**
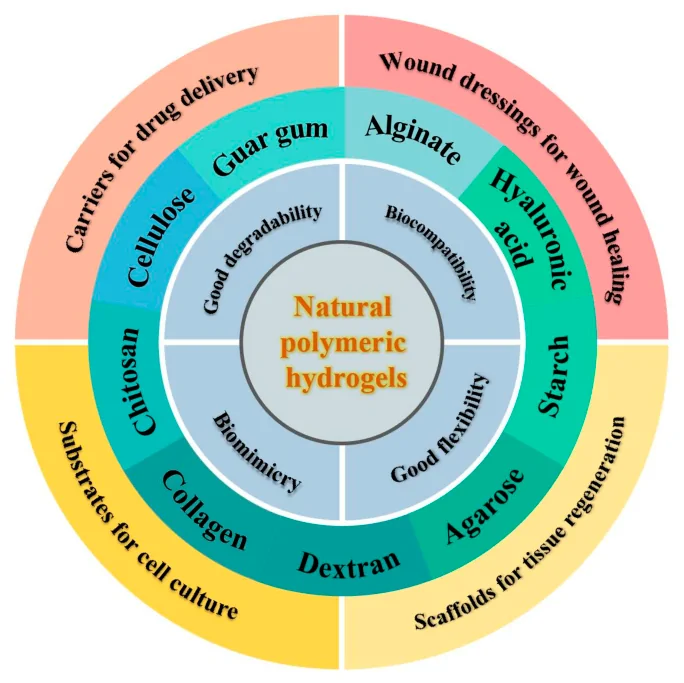
Natural-polymer hydrogel families and their material attributes and biomedical roles, including wound-dressing matrices, drug carriers, and cell-support scaffolds. (Reprinted from Ref. [18]). The colors distinguish polymer families, material attributes, and representative application sectors and do not encode quantitative values.

Matrix location is critical: a polymer that is advantageous at the wound-contact surface may interfere with sensing when placed directly around the recognition element. High absorbency facilitates exudate control, but reservoir volume may dilute or delay the sample before detection [16]. ECM-like protein matrices improve contact and conformity, while their wet stability depends on network structure, crosslinking, and reinforcement [17]. Natural-polymer hydrogels can also combine hydrated wound contact with drug-delivery and cell-support functions [18], providing the material-role overview shown in Figure 1. Wound-healing compatibility should be evaluated separately from the requirements imposed by the sensing domain.

#### 2.1.1. Hydrophilic Polysaccharides: Reservoirs, Soft Interfaces, and Capillary Supports

Alginate, HA, and cellulose-derived matrices support hydrated wound-contact environments in distinct sensing roles. Alginate is commonly used as an exudate-handling or reservoir domain because ionically crosslinked networks provide high fluid uptake, gel formation, and compatibility with drug-loaded or indicator-containing hydrogels [19]. Molecular weight and mannuronic/guluronic-acid composition influence chain entanglement and Ca2+-mediated junction formation, while crosslink density governs a coupled trade-off: denser networks generally improve wet integrity and functional-phase retention but reduce swelling, effective diffusivity, and response speed. Dynamic CMC/OHA/SA/TOB hydrogels and SA/PVA/curcumin hydrogel fibers illustrate how composition and reinforcement can combine antimicrobial delivery, swelling control, and pH indication [20,21]. For sensing, the relevant variables are not only equilibrium uptake but also network-dependent diffusion distance, ion exchange, indicator localization, and the time required for the reservoir to equilibrate with changing exudate.

In sensor-integrated dressings, HA is most useful when the wound interface requires a soft, hydrated transition. HA molecular weight controls solution viscosity, chain relaxation, and degradation, whereas the degree of chemical substitution determines the number of crosslinkable or adhesive groups. Increasing substitution or crosslink density can improve retention and wet-state lifetime but may reduce water uptake and slow analyte transport. HA-rich matrices provide compliant tissue contact between the wound and mechanically dissimilar sensing layers [22,23]. In conductive HA/chitosan hydrogels, Schiff-base bonding, ionic additives, and anti-drying components enable HA to function within a flexible sensing interface [24]. These systems require reporting of molecular weight, substitution, polymer ratio, and wet-state modulus because nominally identical HA formulations can present different transport and deformation histories to the sensor.

**Figure 2:**
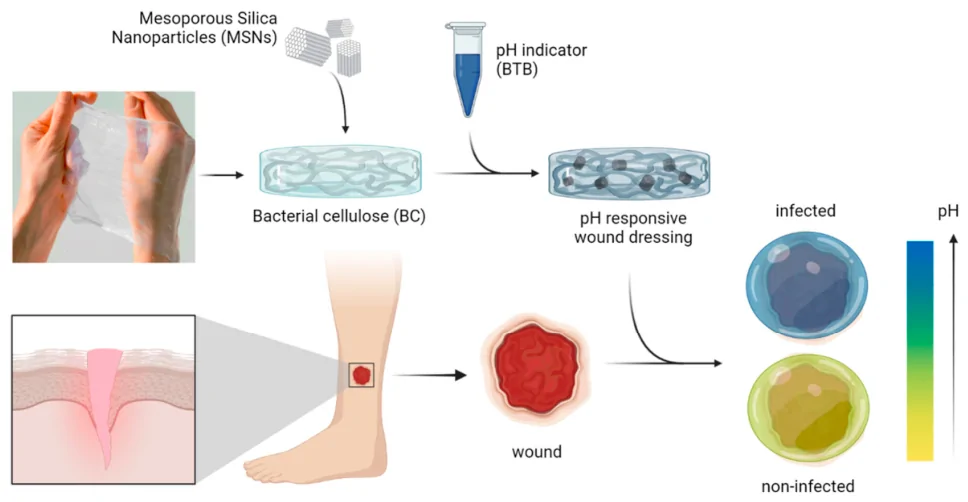
A cellulose-based bacterial-cellulose dressing architecture combines a hydrated polymer host, embedded pH-indicator chemistry, and visual wound-state readout at the wound interface. (Reprinted from Ref. [31]). The arrows indicate the preparation and application sequence, and the pH color scale represents the visual transition between non-infected and infected wound conditions.

Cellulose-derived materials occupy several regions of the dressing architecture. Cellulose, nanocellulose, and bacterial cellulose provide hydrated fibrous support, capillary transport, and optical-sensing compatibility [25,26]. Fiber diameter, fibril packing, porosity, surface oxidation, and composite fraction determine capillary pressure, tortuosity, adsorption, and wet reinforcement. Nanocellulose and bacterial-cellulose matrices have hosted anthocyanin, pH-sensitive, and protease-responsive optical elements [27,28]. Because bacterial cellulose has limited intrinsic antibacterial activity, additional antimicrobial phases are often required [29]. Paper-based and cellulose-derived microfluidic substrates offer low-cost capillary pathways, but nonspecific protein adsorption can alter both flow and recognition [30]. In the BC-MSN-BTB platform, the color transition occurred within seconds over pH 5.5–8.5, remained reversible during pH cycling, and PEI-capped dye retention was maintained during 14 days of water storage; an infected porcine wound produced a visible change in less than 1 min [31] (Figure 2). This example shows why capillary geometry, optical background, wet mechanics, and retention should be reported with sensor response.

#### 2.1.2. Protein-Derived and Cationic Matrices: Contact, Transition, and Active Interfaces

Gelatin and collagen are best treated as tissue-compatible contact or transition materials because they provide extracellular-matrix-like surfaces and hydration-friendly interfaces [32]. Their molecular weight distribution, degree of functionalization, enzymatic susceptibility, and crosslink density jointly determine swelling, wet softening, degradation, and analyte access. Silk fibroin provides additional processing-dependent mechanical tunability and structural stability [33], while multifunctional silk-fibroin hydrogels integrate antibacterial and regenerative functions [34]. PLA-nanofiber reinforcement of methacrylated gelatin composites and spatially defined dye-containing layers in infection-responsive collagen fibers offer complementary routes to preserving wet-state structure and localizing optical chemistry [35,36]. Quantitative comparison remains difficult because many studies report dry mechanics or equilibrium swelling without the paired wet-state response time and functional-phase retention needed for sensing interpretation.

**Figure 3:**
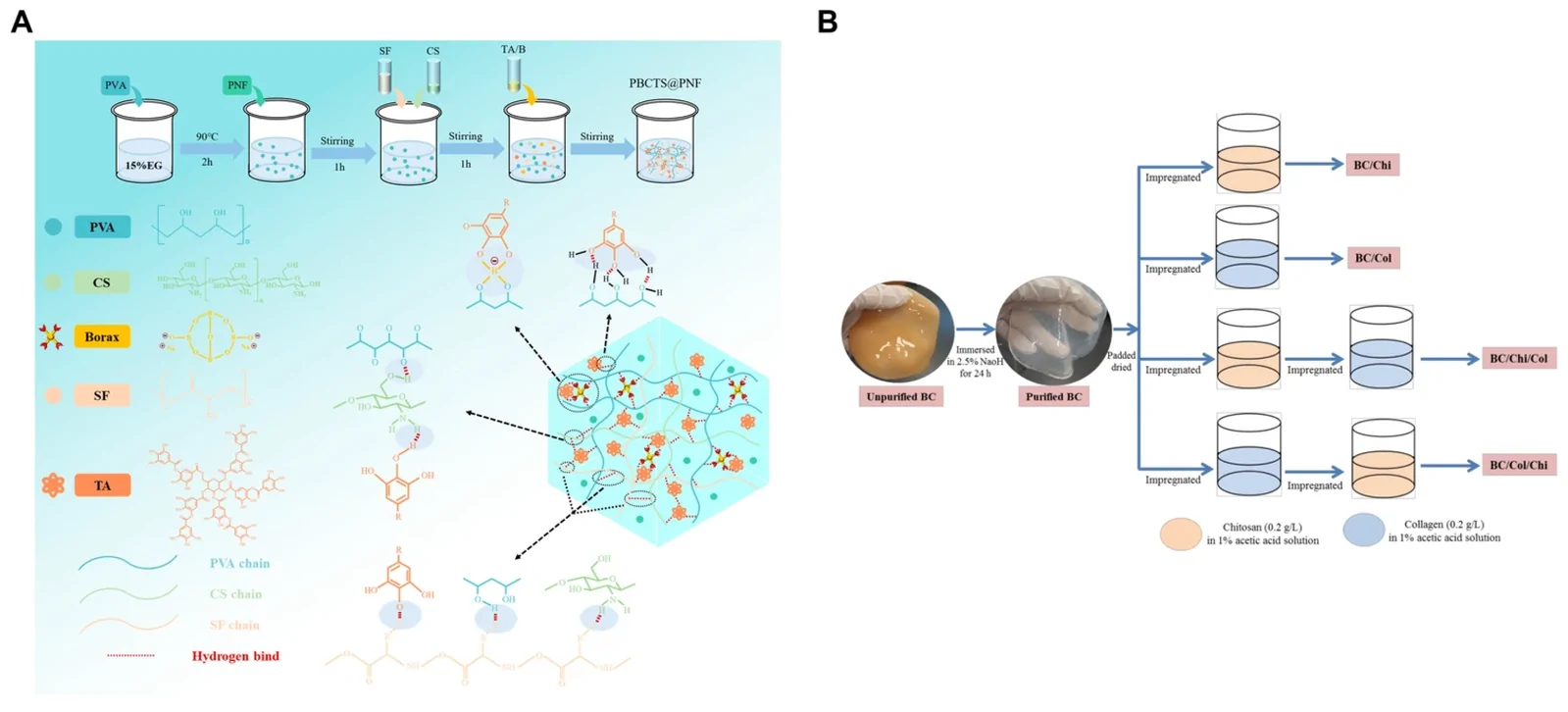
Protein-derived and cationic biopolymer interfaces for wound-contact matrices. (**A**) Silk-fibroin-containing hydrogel formation uses multicomponent polymer mixing and network-forming interactions to generate a hydrated matrix. (Reprinted from Ref. [34]) (**B**) Bacterial cellulose is sequentially impregnated with chitosan and collagen to tune dressing composition and interfacial chemistry. (Reprinted from Ref. [41]). Arrows indicate the preparation sequence; colors distinguish material components and do not encode quantitative values.

Chitosan exhibits stronger interfacial interactions than many neutral or weakly charged natural polymers. Its amino groups contribute to hemostasis and wound repair [37,38], while molecular weight, degree of deacetylation, and chemical substitution control charge density, solubility, chain mobility, adsorption, and crosslinking. These variables can alter protein fouling and the partitioning of charged analytes at a sensing boundary. Molecular-weight-dependent keratinocyte effects and structurally distinct macrophage responses show that increasing cationic character is not a uniformly beneficial design change [39,40]. Chitosan is most interpretable when confined to a defined wound-contact, antimicrobial, or immobilization domain. Sequentially impregnated bacterial-cellulose/chitosan/collagen mats illustrate how cationic and protein-derived components can be organized into distinct interfacial regions [41] (Figure 3).

#### 2.1.3. Auxiliary Functional Phases in Biobased Composite Dressings

Most biobased matrices lack intrinsic conductivity, catalytic activity, strong optical response, or highly specific biochemical recognition. Auxiliary phases supply these functions, but polymer-to-additive ratio, particle dispersion, percolation threshold, and immobilization chemistry determine whether the added function survives hydration [42]. PVA can regulate processing, toughness, and water management. Conducting polymers create flexible electrochemical pathways, although hydrolysis and dopant loss may impair prolonged wet operation [43]. Carbon and MXene networks depend on percolation pathways that can shift as the matrix swells; MXenes also face oxidation and hydrolysis in aqueous environments [44,45]. Silver-containing phases require a balance between antibacterial release and cytotoxic exposure [46,47]. Composition should be reported as a coupled network variable, not only as an ingredient list.

**Figure 4:**
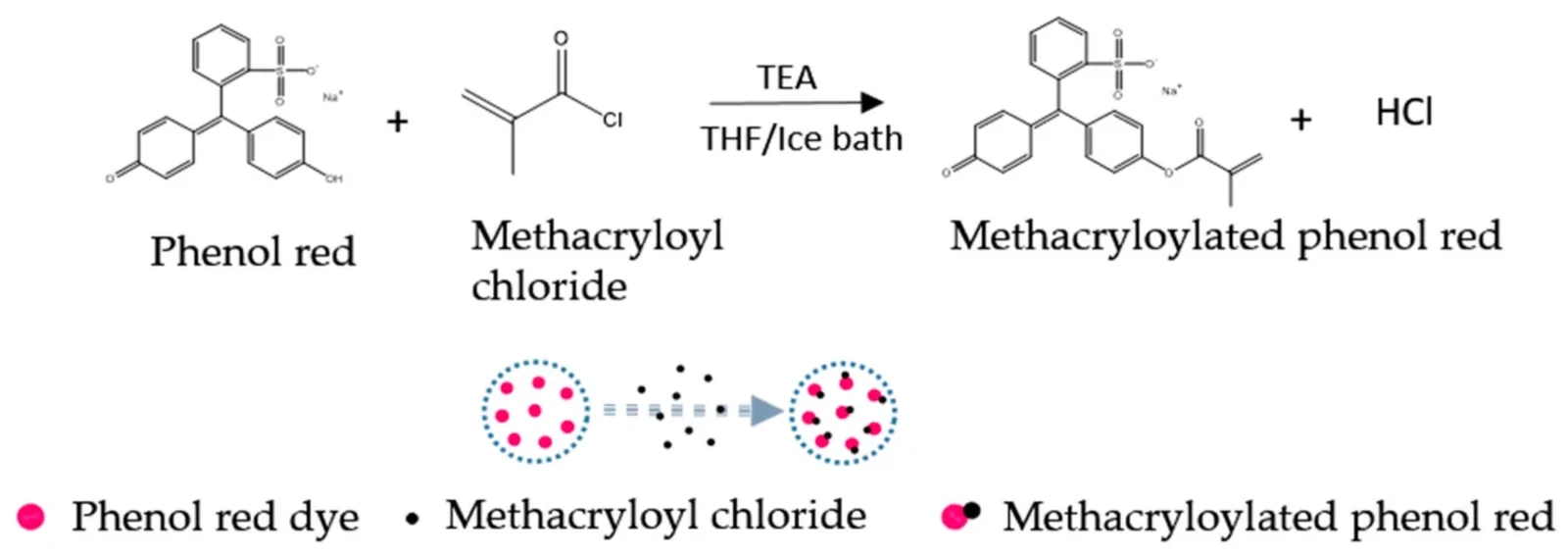
Methacrylated phenol red as an auxiliary functional phase for optical pH sensing. Methacrylation provides a chemical route for covalently incorporating the indicator into a hydrogel network. (Adapted from Ref. [50]).

Biochemical sensing raises a retention problem. During immersion, repeated switching, or exposure to wound-like fluid, a pH or infection indicator that diffuses out no longer reports a defined location [48]. In cellulose-chitosan hydrogels containing polydiacetylene/ZnO nanosheets, responsive and antibacterial components are organized throughout the matrix instead of introduced as freely dispersed substances [49]. Methacrylated phenol red provides a chemical route for preparing pH-responsive chemistry for subsequent covalent integration into a hydrogel network [50] (Figure 4). Enzymes and antibodies require immobilization chemistries that preserve activity while keeping recognition sites accessible in hydrated, protein-rich samples. Performance is determined by whether the functional phase remains spatially stable, accessible, and compatible with the surrounding matrix.

Even when a colorimetric dye is retained, a swollen network may delay its response by increasing diffusion length or reducing effective diffusivity. Immobilized enzymes face the same transport-activity trade-off, whereas conductive fillers can lose pathway continuity during swelling, oxidation, or fatigue [51]. Crosslink density and functional-phase loading are non-monotonic design variables: increasing either may improve retention or signal magnitude while slowing transport, increasing stiffness, or reducing accessible active area. Meaningful optimization requires paired measurements of swelling or water uptake, wet mechanics, response/recovery time, retained activity, and signal stability under the same exposure conditions.

#### 2.1.4. Material-Role Map

Table 2 compares molecular and network variables with matched wet-state and sensing quantities from representative systems. The values are not pooled because test media, specimen geometry, conditioning time, and readout definitions differ. They are presented to show the scale of reported behavior and to prevent qualitative terms such as high swelling, stable, or rapid from being treated as equivalent.

The numerical examples reveal points that a polymer-by-polymer description alone obscures. The SA/PVA/CUR fiber reached 266% swelling, but the sensing-response time remained unreported [21]. An HA/chitosan hydrogel achieved a gauge factor of 2.64, yet it was evaluated as a strain sensor instead of for wound-fluid chemistry [24]. Collagen fibers combined 323–492% swelling with 12–15 MPa wet tensile strength and retained most of their dye over 72 h [35]. Conditioning history can also dominate composition: drying shifted the modulus of an alginate/PAAm indicator patch from the sub-MPa range to 155 MPa, and re-swelling did not restore the original state [50]. Cross-study ranking is not justified because conditions differ, but the comparison shows why molecular specification, hydration history, wet mechanics, and sensing response must be reported together.

### 2.2. Exudate Routing and Controlled Sensing-Zone Exposure

In sensor-integrated wound dressings, exudate-handling architecture controls how wound fluid reaches the sensing region. Beyond maintaining the wound environment, this architecture determines sample volume, residence time, renewal rate, and the exposure history of the sensing interface. Directional dressings, microfluidic pathways, and absorbent-layer-driven systems illustrate how drainage direction, chamber volume, and sensor-window placement alter the timing and quantity of fluid delivered to the readout region. Exudate routing is thus a functional component of the sensing architecture because it determines the fluid history presented to the readout region.

**Figure 5:**
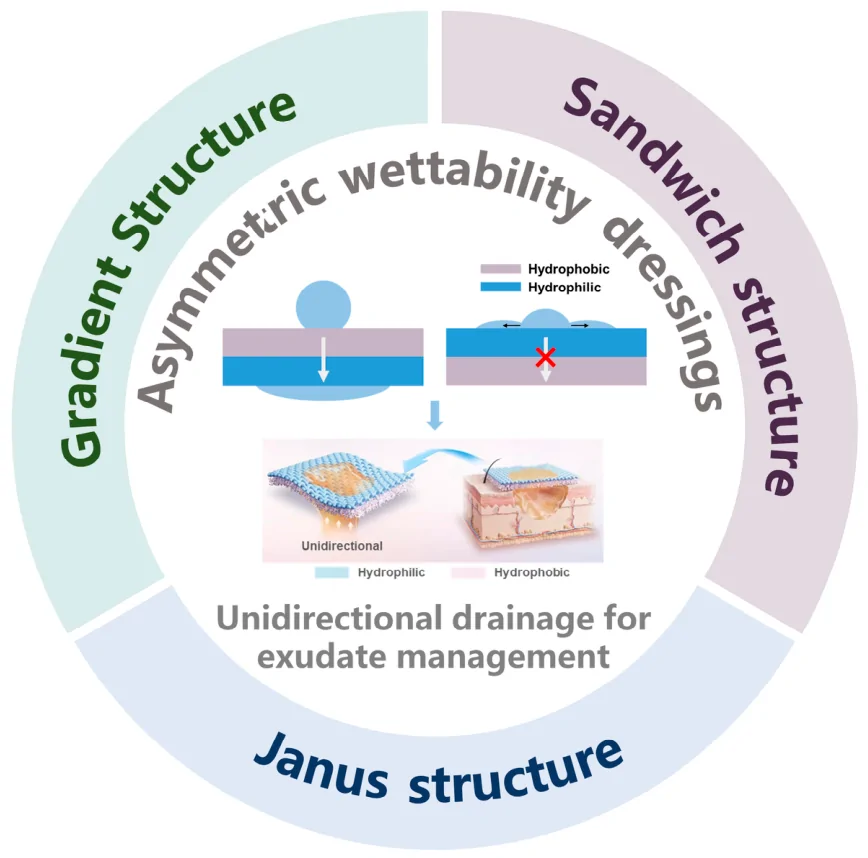
Asymmetric-wettability architectures for wound-exudate management. Janus, sandwich, and gradient structures create spatial differences in wettability and capillary pressure that direct exudate away from the wound-facing side. (Reprinted from Ref. [52]). Arrows indicate the intended direction of fluid transport; colors distinguish Janus, sandwich, and gradient architectures.

Existing platforms can be grouped according to four main routing functions: contact-side fluid entry, absorbent or capillary storage, directional partitioning, and controlled sensing-zone exposure. These routing modes impose different sampling conditions. Although reservoir domains can stabilize local moisture conditions, they may delay or temporally average changes in analyte concentration. Janus or capillary layers may promote more effective sample renewal, but they remain susceptible to fouling or blockage. Microfluidic channels can provide defined sample volumes, while introducing deformation-, leakage-, or filling-related errors. Comparisons among these systems are most informative when sensor response is reported together with the fluid-transport history that produced it. Asymmetric-wettability dressings organize these pathways through Janus, sandwich, or gradient architectures that regulate unidirectional transport and fluid redistribution [52] (Figure 5).

#### 2.2.1. Contact-Side Entry and Upstream Conditioning

Soft natural matrices, including gelatin, collagen, hyaluronic acid, bacterial cellulose, chitosan, and alginate, are commonly selected because they support hydration and reduce contact-related tissue damage [53]. Gelatin/HA-assembled hydrogels and systems with switchable adhesion demonstrate how antibacterial function and low-trauma removal can be combined at the same interface [54,55]. These wound-care characteristics do not fully describe the sensing role of the same layer. Before exudate reaches a downstream sensor, contact-side swelling, charge, degradation, and protein affinity may already have altered its composition or transport history.

The matrix may therefore be well tolerated by tissue without being analytically neutral. Chitosan at the contact surface can support hemostasis and microbial control while simultaneously altering protein adsorption, background pH, and cellular activity. Highly swollen domains may protect the wound yet retain or dilute soluble analytes and delay their arrival at the sensor. Evaluation of the contact layer requires separate criteria for wound-care performance and sample collection. Moisture balance, wet contact, and removal trauma describe wound-care performance; inlet area, uptake kinetics, adsorption, prefiltration, and delay describe sample collection. Janus membranes, wedge-shaped channels, and graded micropillars in microfluidic exudate-management devices demonstrate how these sampling variables can be deliberately controlled at the wound interface [56].

#### 2.2.2. Absorbent Reservoirs and Capillary Substrates

**Figure 6:**
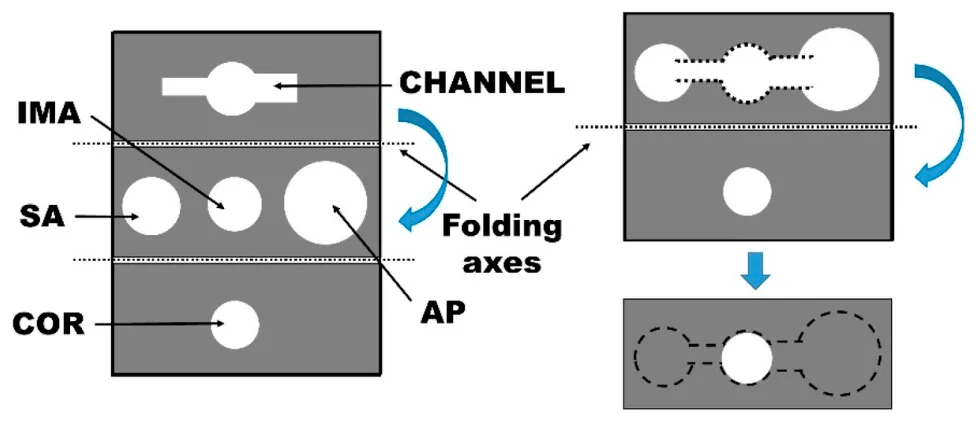
Foldable paper-based capillary device for wound-fluid analysis. Sample, antibody-immobilization, absorbent, and chromogenic regions are integrated into a patterned cellulose substrate to direct fluid toward a colorimetric infection readout. (Reprinted from Ref. [62]). Arrows indicate the folding sequence and alignment of the sample, conjugate, and test regions.

Absorbency controls the amount of exudate retained by the dressing but does not by itself define a useful sample pathway. Commercial dressings differ in swelling, fluid retention, moisture-vapor transmission, and wet-state mechanics [57]. In hydrogel systems, pore structure and exudate viscosity determine the extent and uniformity of penetration [58]. Cellulose paper devices provide more explicit control: patterned channels guide capillary flow [59,60], Janus papers impose directional uptake while delivering antibiotics [61], and foldable devices guide fluid through collection, recognition, absorbent, and chromogenic regions [62] (Figure 6). Water or buffered saline cannot reproduce the viscosity, adsorption, and blockage produced by proteins, cells, blood components, and bacteria. Capillary performance should be tested with wound-like fluids spanning the intended viscosity and fouling range.

A compact transport framework helps connect architecture with sensor response. A 2025 porous-dressing study provides a quantitative bridge between structure and transport: cellulose dressings showed at least 48% greater wettability/imbibition performance than alginate or polyester comparators, while increased exudate viscosity and glucose content prolonged absorption time by approximately 51% and 38%, respectively [63]. For diffusion over a characteristic distance L, the equilibration time scales as tD approximately L^2/Deff, where Deff is reduced by tortuosity, binding, and network obstruction. Convection becomes increasingly important as the Peclet number Pe = UL/Deff rises, where U is the mean fluid velocity. In a capillary pore of radius r, the driving pressure scales as Delta P = 2 gamma cos(theta)/r, and the Lucas–Washburn relation predicts l^2 proportional to gamma r cos(theta)t/(2 mu); higher viscosity mu or unfavorable contact angle theta slows filling. These relations explain why thicker swollen layers, narrow or fouled pores, and high-viscosity exudate can increase response time even when the sensing chemistry is unchanged.

Partitioning and adsorption further separate the concentration at the sensor from that in bulk exudate. A partition coefficient between fluid and polymer defines the equilibrium concentration entering the matrix, while reversible adsorption retards transport and irreversible adsorption reduces analyte recovery. Charged chitosan, oxidized cellulose, proteins, dyes, and nanoparticles can each alter this balance. Transport studies should report L, thickness or pore scale, fluid viscosity, inlet area, filling time, retained volume, flow rate when applicable, and recovery of a known analyte concentration. Without these variables, differences in signal amplitude or lag cannot be assigned confidently to recognition chemistry, fluid routing, or matrix retention.

#### 2.2.3. Directional, Janus, and Microfluidic Partitioning

**Figure 7:**
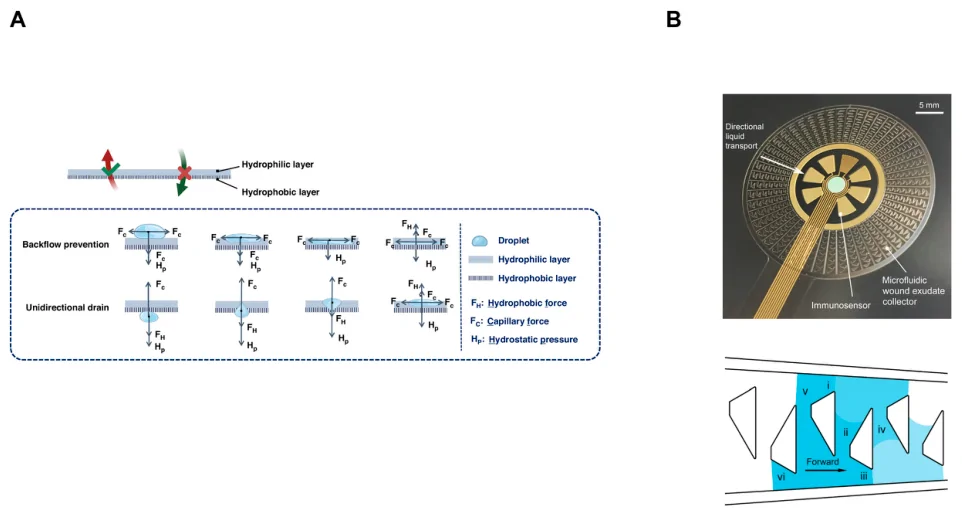
Directional exudate routing and microfluidic sample presentation in smart wound dressings. (**A**) Liquid-diode structures use asymmetric wettability to promote one-way drainage and reduce backflow. (Adapted from Ref. [68]) (**B**) Microfluidic wound-exudate collectors position directional transport and immunosensing elements at the wound interface. (Adapted from Ref. [69]). In panel (**B**), Roman numerals i-vi mark successive liquid-front positions through the asymmetric micropillar channel, illustrating forward transport.

Directional transport systems are built to reduce random wetting by controlling the direction and location of exudate exposure. Hydrophobic-to-hydrophilic gradients and self-pumping nanofiber structures can reduce rewetting and convert passive fluid uptake into unidirectional transport [64]. (Polylactic acid, PLA)/(Polyacrylonitrile, PAN) Janus nanofiber dressings and cellulose-based pH-responsive Janus dressings further show how asymmetric drainage can be combined with monitoring or antibacterial functions [65,66]. Xiang et al. extended this principle through a battery-free microfluidic Janus dressing that integrates unidirectional fluid transport, antibacterial surface treatment, colorimetric sensing, and smartphone-assisted recognition of pH, uric acid, and temperature [67]. Liquid-diode smart bandages also use one-way exudate drainage to support dynamic pH sensing [68]. Microfluidic and textile-based platforms provide another level of routing control by defining chamber volume, flow path, and sensor residence time. Flexible multiplexed immunosensor layouts further show how wound-exudate collectors can position sampling pathways and sensing windows at the wound interface [69] (Figure 7). Across these formats, polymer architecture identifies the variables that control sampling before signal conversion, including inlet geometry, channel volume, absorbent driving force, sample renewal, ionic contact, and exposure stability.

#### 2.2.4. Routing Failure in Multilayer Platforms

Multilayer dressings spatially separate wound contact, fluid handling, exudate routing, sensing, and mechanical support into different functional domains. This compartmentalized architecture can reduce lateral leakage, backflow, uncontrolled sensor immersion, and interference among integrated functions. Bypass flow, channel collapse, interlayer delamination, fluid pooling, sensing-window misalignment, and localized drying may change the volume, composition, or timing of the sample delivered to the readout region without changing the intrinsic sensing chemistry. Studies of flexible microfluidic systems have further shown that channel deformation directly alters fluid transport and device performance [70]. Material architecture should be related to quantitative sample-delivery metrics. Relevant endpoints include filling time, dead volume, retained fluid volume, leakage, backflow, sensor-window contact area, flow stability after mechanical deformation, and signal recovery after wet exposure. Inadequate absorption, retention, or redistribution can cause fluid pooling and leakage, leading to peri-wound maceration and additional clinical burden [71,72]. These parameters also show that the wound-contact interface, fouling environment, and associated therapeutic functions can continue to alter the readout after exudate reaches the sensing zone.

### 2.3. Wound-Interface Functions as Sensing Conditions

The wound-contact interface does more than provide a biologically compatible surface for tissue repair. In sensor-integrated wound dressings, it also conditions the wound-fluid sample and the sensing boundary. Exudate volume, protein content, and bacterial contamination can affect sample renewal, electrode fouling, and optical background, consistent with recent discussions of wearable wound biosensors [73]. The wound-interface literature should be interpreted from two complementary perspectives: wound-care functions determine whether a dressing remains clinically suitable, while the same interfacial properties determine whether the analytical signal is generated under stable and interpretable conditions.

#### 2.3.1. Exudate Management and Matrix Conditioning

**Figure 8:**
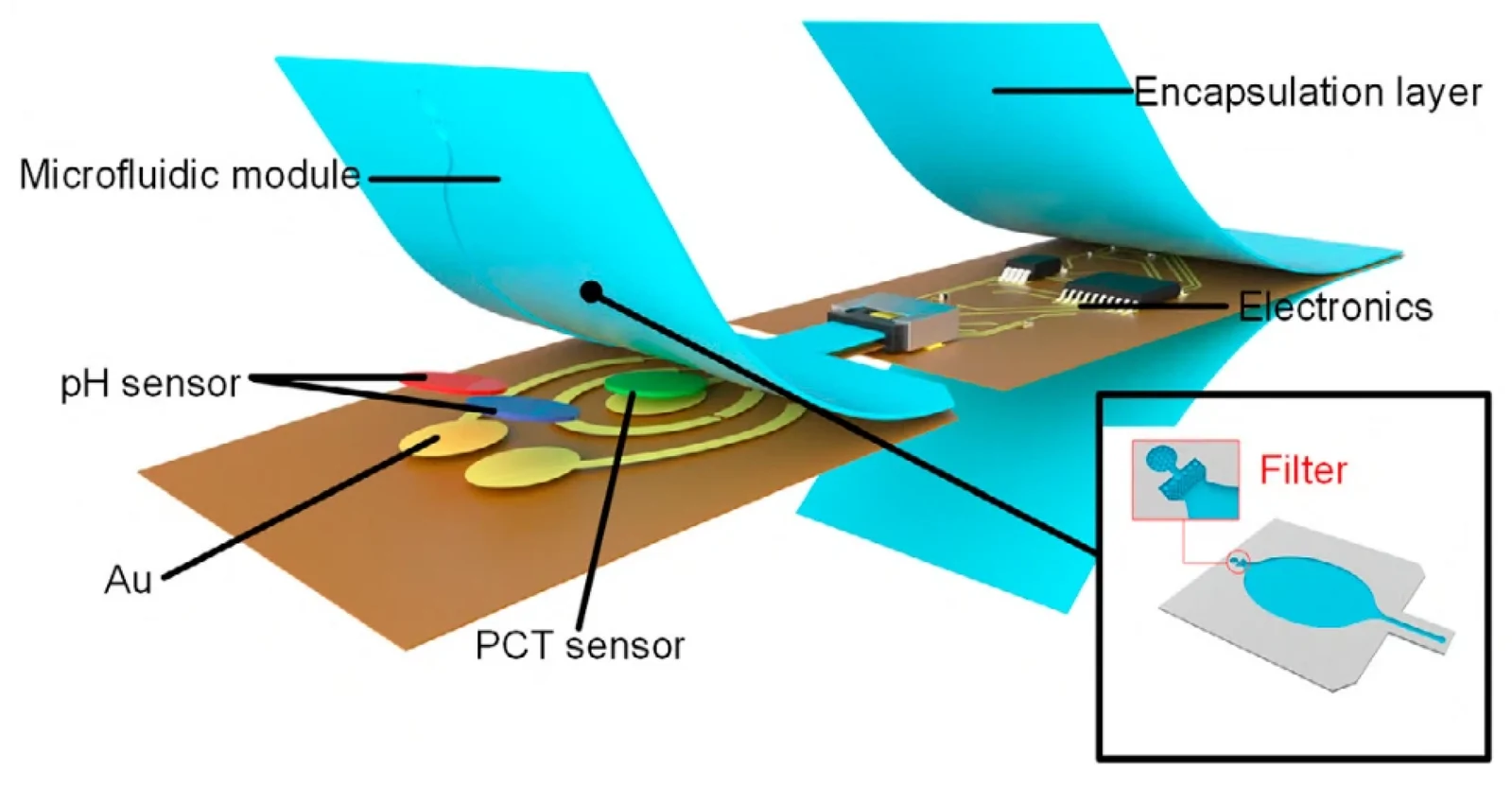
Battery-free wound-dressing system for conditioned sample delivery and wireless sensing. The layered architecture combines a microfluidic module and filter with pH and procalcitonin sensors, encapsulation, and electronics. (Adapted from Ref. [74]).

Exudate management is a wound-care function because excessive fluid accumulation can lead to dressing saturation, leakage, peri-wound maceration, and increased patient burden. In sensor-integrated wound dressings, however, the same fluid-handling properties also define the analytical state in which the sample is presented to the sensing interface. Fluid loading, viscosity, retention capacity, vapor transmission, and wet-state mechanical behavior determine whether the sensing window is exposed to freshly renewed exudate, a diluted reservoir sample, or a partly filtered and temporally aged fluid fraction. The battery-free platform integrating microfluidic collection, contaminant filtration, and pH, temperature, and procalcitonin sensing shows how exudate handling can act as a sample-conditioning step before detection [74] (Figure 8). Absorbency should not be considered only a determinant of patient comfort or moisture balance; it also controls sample residence time, dilution, renewal, and the matrix-derived background encountered by the sensor.

#### 2.3.2. Antibacterial and Antifouling Interfaces

**Figure 9:**
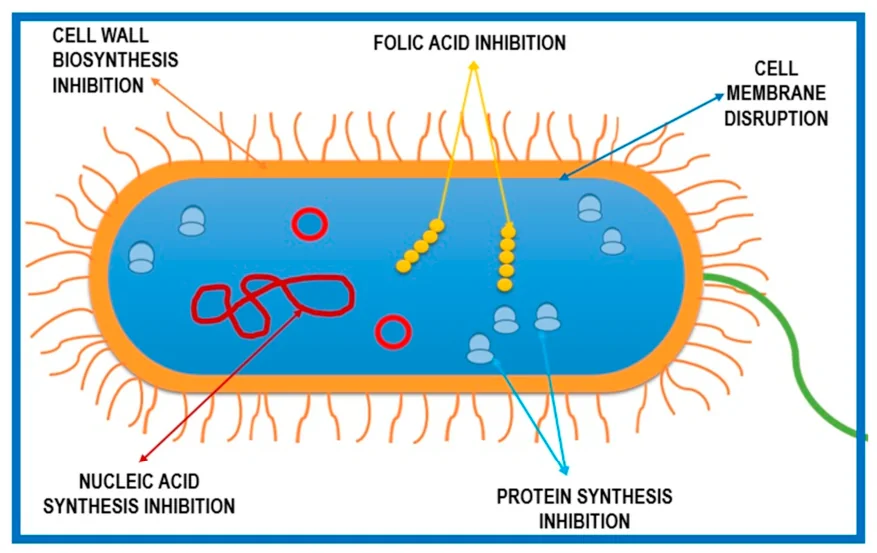
Major cellular targets of antibacterial agents relevant to wound-contact material design. The source schematic summarizes membrane disruption and inhibition of cell-wall, folate, nucleic-acid, and protein-synthesis pathways. (Reprinted from Ref. [76]).

Antibacterial functionality is an important consideration in wound-contact material design, but it is not analytically neutral in sensor-integrated dressings. It is particularly relevant to biofilm-associated wounds, where persistent microbial communities maintain inflammation and complicate clinical assessment [75]. Metal nanoparticles and photothermal materials act through ion release, reactive oxygen species, local heating, or combinations of these effects [8]. Other antibacterial strategies target cell membranes, cell walls, folate pathways, nucleic acids, or protein synthesis (Figure 9). Chitosan is associated with electrostatic membrane disruption, intracellular interference, and chelation [76]. These interfacial effects may reduce microbial burden while changing the local chemical environment presented to nearby sensors.

Antifouling protection is directly related to signal reliability. Wound exudate contains proteins, blood components, cellular debris, bacteria, and biofilm residues that may shield electrodes, mask recognition elements, or alter optical backgrounds. Electrochemical biosensing studies therefore emphasize the need for antifouling interfaces in surface-based detection systems [77]. The (oxidized bacterial cellulose, OxBC)/(quaternized chitosan, QCS) electrochemical biosensor provides a useful example because its bacterial-cellulose/chitosan interface was evaluated not only as a wound-compatible hydrogel, but also as an antifouling and antibody-immobilizing layer for involucrin detection in wound exudate [78]. In general, antibacterial activity and antifouling performance should be evaluated as separate interfacial functions.

#### 2.3.3. Contact Compatibility, Immobilization, and Mechanical Stability

Compatible wound contact can reduce irritation, dressing-change pain, and material-induced biological background. Studies of atraumatic dressings have linked softer contact interfaces to reduced pain and stress during dressing removal [79]. Adhesion-switchable and bioinspired hydrogels further show that wet adhesion, low-trauma removal, pH monitoring, and antibacterial activity can be integrated within a single dressing interface. From the perspective of sensing interpretation, however, a high density of functions at the wound-contact surface is not necessarily beneficial. When adhesion, antimicrobial activity, and sensing chemistry occupy the same hydrated domain, the resulting readout may reflect not only wound status, but also local swelling, pH buffering, or material release. Material-driven biological background should be treated as part of the sensing challenge, not only as a biocompatibility issue.

Functional-phase immobilization links material design to sensor reliability at the interface. For an optical pH sensor, a defined exposure window and experimental evidence are needed to establish that the dye remains localized during hydration; dye-retention measurements make leakage directly visible [80]. Enzyme- and antibody-based sensors must preserve catalytic or binding function while allowing the relevant analytes to reach immobilized sites. Zwitterionic enzyme coatings provide one approach to maintaining this balance in electrochemical biosensing [81]. Ionically conductive hydrogels introduce a related constraint: swelling, adhesion, and signal repeatability must remain compatible during cyclic deformation [82].

#### 2.3.4. Therapy–Monitoring Coupling

**Figure 10:**
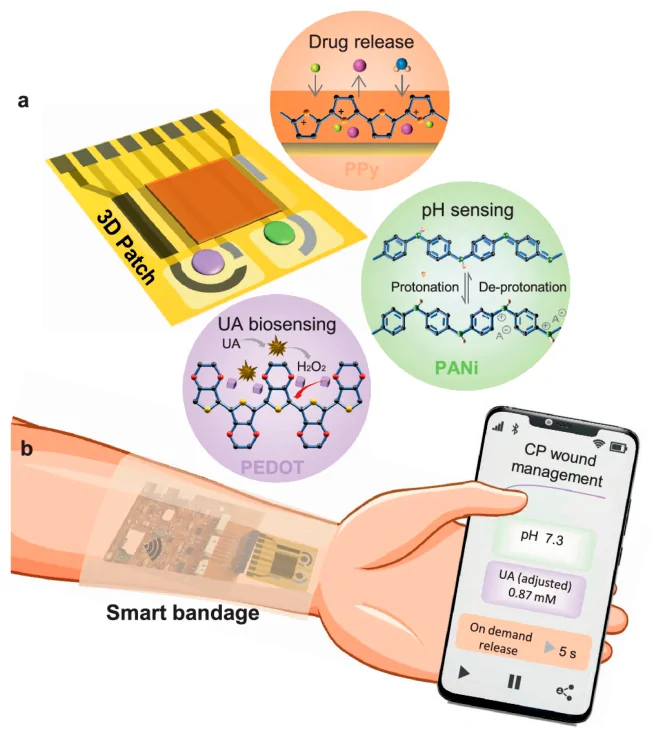
Therapy-monitoring coupling in conducting-polymer smart bandages. (**a**) A 3D-printed patch integrates polypyrrole (PPy)-based drug release, polyaniline (PANi)-based pH sensing, and poly(3,4-ethylenedioxythiophene) (PEDOT)-based uric-acid biosensing. (**b**) The skin-mounted smart bandage connects these functions to smartphone readout and on-demand drug release. (Reprinted from Ref. [85]). Colors distinguish the sensing, drug-release, and conducting-polymer components and do not encode quantitative values.

In therapeutic and diagnostic dressings, treatment and sensing are particularly difficult to separate because both processes occur within the same small fluid volume. The alginate/graphene oxide/levofloxacin/silk system combines pH indication with pH-responsive drug release [83]. NIR-triggered microneedle patches combine pH monitoring with photothermal or chemodynamic antibacterial action [84], whereas conducting-polymer bandages integrate pH sensing, pH-compensated uric acid detection, and electrically triggered ciprofloxacin release [85] (Figure 10). These configurations are attractive precisely because their modules interact locally. These same interactions also prevent therapy and monitoring from being interpreted as independent modules once they share temperature, pH, ionic conditions, or a colorimetric background.

Therapy-monitoring coupling is most convincingly evaluated by separating sensor-only, treatment-only, blank-material, and fully integrated controls and by confirming after use that sensing and therapeutic modules remain physically intact and functionally distinct [86,87]. For material interpretation, the critical issue is whether treatment alters the monitored variable, the sensing boundary, or the conditions under which the signal is generated. These interactions determine which material limitations must be considered for each readout class.

### 2.4. Readout Modalities and Material-Dependent Constraints

**Figure 11:**
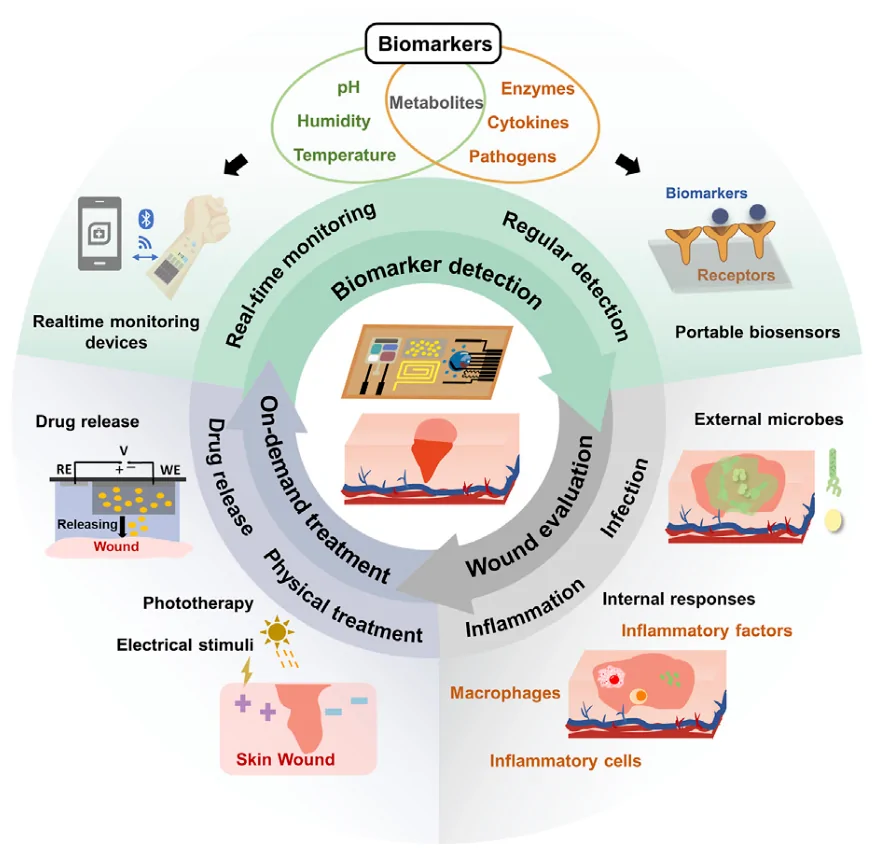
Material-to-readout organization in conformable wound-management systems. Wound biomarkers are coupled to portable or real-time sensing, infection and inflammatory-state evaluation, and on-demand physical or drug-based treatment within a shared wound-interface platform. (Reprinted from Ref. [89]). Arrows indicate relationships among biomarkers, monitoring modalities, and therapeutic functions; colors distinguish functional categories.

Wound-exudate sensors are commonly grouped by target analyte, but their material requirements are more clearly differentiated by the mechanism of signal generation and the matrix variables that influence that mechanism. Optical pH indicators, electrochemical metabolite sensors, immunochemical and protease-responsive systems, and multiparameter platforms rely on different matrix hosts, fluid-contact geometries, immobilization strategies, and calibration media [88,89]. The supporting evidence also varies: some studies report colorimetric or electrochemical responses in controlled buffers, whereas others incorporate simulated wound fluid, dynamic flow, real or ex vivo exudate, wireless acquisition, or treatment-coupled monitoring. These experimental settings probe different sources of error and therefore require readout-specific material controls. Figure 11 summarizes this material-to-device continuum.

#### 2.4.1. pH and Optical/Electrochemical Readouts

pH remains one of the most widely developed readouts in sensor-integrated wound dressings because it can be monitored through relatively simple optical or electrochemical interfaces. Existing systems generally follow two main material strategies. In optical formats, the polymer matrix functions as a hydrated colorimetric window or fluorescence host, as demonstrated by bicolorimetric chitosan hydrogels, chitosan/alginate pH-responsive hydrogels, and carbon-dot-containing nanofiber systems [90,91,92]. In electrochemical formats, reliable readout depends primarily on hydrated electrode contact, stability of the pH-sensitive film, and ion accessibility [93]. In both approaches, the matrix remains at the center of signal generation instead of serving only as a passive support substrate.

**Figure 12:**
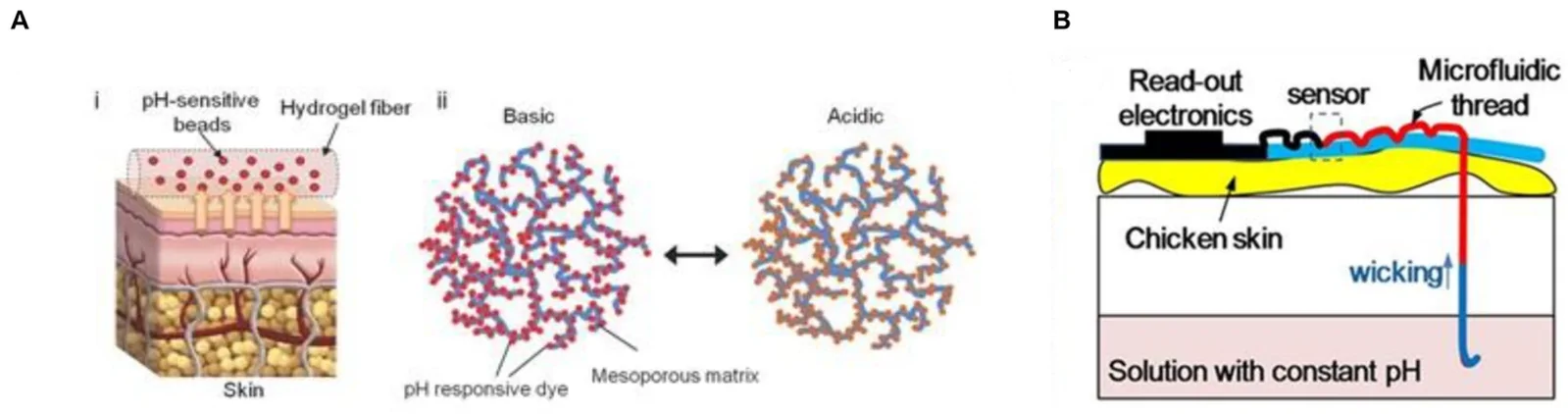
Complementary pH-sensing routes in wound-contact systems. (**A**) pH-responsive dye is retained in mesoporous particles embedded within alginate hydrogel fibers. (Adapted with permission from Ref. [96]. Copyright 2016 WILEY-VCH Verlag GmbH & Co. KGaA, Weinheim.) (**B**) Wound fluid is wicked through a thread-based microfluidic pH sensor toward external readout electronics. (Adapted from Ref. [97]). In panel (**A**), (i) depicts the pH-sensing hydrogel-microfiber architecture and (ii) depicts the mesoporous-particle/dye response mechanism; arrows indicate the direction of sample transport or response.

The major material limitations vary with the readout format. Optical pH systems are particularly sensitive to dye leaching, matrix thickness, optical background, swelling-induced changes in optical path length, and image-acquisition conditions. Smartphone-based and deep-learning-assisted colorimetry may improve image interpretation [94,95], whereas dye retention, staining, protein deposition, and sensing-window thickness still require separate material controls. Electrochemical pH systems shift the major concern to hydrated electrode contact, signal drift after wet aging, and recovery after repeated exposure. Theranostic pH dressings add another layer of complexity because drug release, photothermal treatment, or antibacterial chemistry may interfere with the same pH environment or optical background being monitored. Complementary matrix- and fluidic-based strategies are illustrated by mesoporous dye-loaded hydrogel fibers and thread-based microfluidic pH-sensing systems [96,97] (Figure 12).

#### 2.4.2. Metabolites, Uric Acid, and Reactive Species

**Figure 13:**
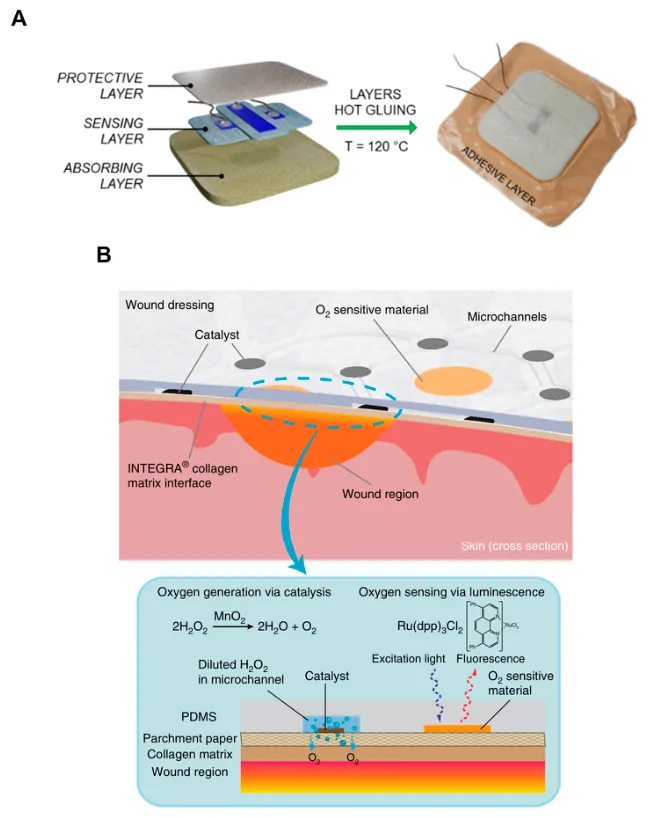
Wearable architectures for metabolite and reactive-species monitoring. (**A**) A layered textile organic electrochemical transistor dressing integrates protective, sensing, absorbing, and adhesive domains for uric-acid monitoring. (Adapted from Ref. [98]). (**B**) A wound-contact patch couples catalytic oxygen generation to luminescence-based oxygen sensing through a layered collagen/microchannel architecture. (Adapted from Ref. [100]). Arrows indicate the transport and sensing pathway; colors distinguish device components and signal channels.

Metabolite and reactive-species readouts generally depend more on transport conditions than optical pH windows because the target species often interacts with an enzyme, electrode, or reactive film near the sensing surface. Uric acid monitoring with textile organic electrochemical transistors (OECTs) is especially informative because the sensor response was evaluated under synthetic wound-exudate flow instead of only under static buffer conditions [98]. Printed OECT biosensors with uricase immobilized in a dual-ionic-layer hydrogel further demonstrate that enzyme retention, substrate diffusion, and catalytic conversion must be maintained together in artificial wound exudate [99]. Microfluidic oxygen-sensing wound-dressing systems extend this issue to device-level sampling by coupling local oxygen measurement with controlled fluid delivery and wound-interface operation [100]. These examples illustrate two routes for spatially separating recognition, transport, and readout within wearable dressings (Figure 13).

For these readouts, a low detection limit has limited value if sample renewal, enzyme or electrode accessibility, and post-use baseline stability are not maintained. Enzyme-based signals depend on substrate diffusion, oxygen or cofactor availability, and transport of products away from the recognition zone. Electrode-based signals require ionic access and antifouling protection. Conductive polymers, carbon networks, and MXene-containing phases can enhance signal transduction; however, their effectiveness is determined by whether the hydrated matrix maintains percolation, electrode contact, and aqueous stability during swelling, bending, or deformation. Accordingly, evaluation should address the coupled fluidic and electrochemical behavior instead of electrode chemistry alone.

#### 2.4.3. Protease, Inflammatory, and Infection-Related Markers

Protease, inflammatory, and infection-related markers are detected in a particularly complex material environment because recognition usually occurs in protein-rich, bacteria-containing fluids with substantial optical and electrochemical background interference. Matrix metallopeptidase 9 (MMP-9)-responsive smart dressings illustrate this challenge by combining wireless sensing, nanoparticle-mediated antibacterial release, and comparison with wound exudate [101]. DNase-responsive DNA-hydrogel sensors provide a more infection-specific material strategy by converting nuclease activity from bacteria-rich wound environments into a wireless dielectric readout [102]. Origami-fluidic paper electrochemical devices provide a complementary approach by directing wound exudate to an antibody-functionalized electrode for smartphone-readable myeloperoxidase detection [103].

The evidence gradient is particularly important for these biomarkers. Responses obtained in buffer solutions and simulated wound fluids do not provide the same level of biological interpretation because proteins, blood components, bacterial debris, and biofilm residues can mask recognition sites, block porous substrates, or alter optical and electrochemical baselines. Measurements using real or ex vivo exudate provide a stronger biological context for wound-biomarker platforms. Infection-responsive nanofibrous hydrogel dressings further show how diagnostic response and infection control can be combined within defined material architecture [104]. Protease or inflammatory readouts may nevertheless reflect several biological processes, including infection, delayed repair, tissue degradation, or treatment response. Material design can reduce nonspecific adsorption and preserve sample identity, but biological interpretation still benefits from independent assays or clinical reference measurements.

#### 2.4.4. Multiparameter, Wireless, and Smartphone Readouts

**Figure 14:**
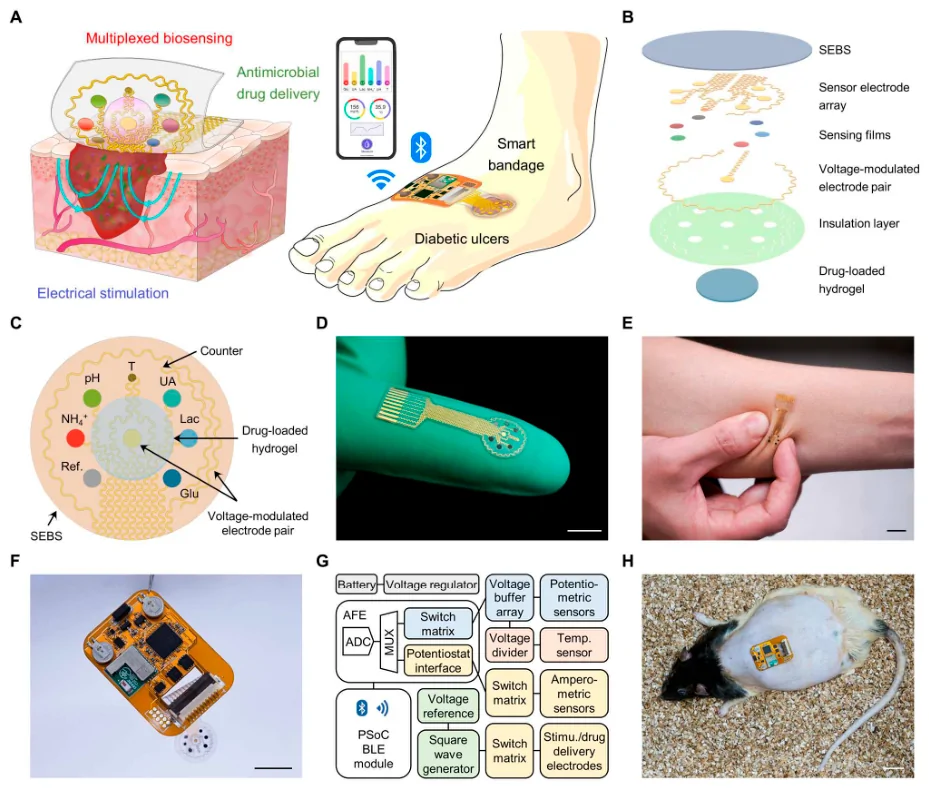
Multiparameter wireless bioelectronic system for wound monitoring and treatment. (**A**) A stretchable smart bandage combines multiplexed biosensing, antimicrobial drug delivery, electrical stimulation, and wireless readout. (**B**,**C**) The architecture separates the substrate, electrode array, sensing films, insulation layer, and drug-loaded hydrogel. (**D**,**E**) Photographs show the flexibility and skin compatibility of the system. (**F**,**G**) Electronic hardware enables wireless acquisition and actuation. (**H**) Preclinical placement demonstrates deployment at the wound interface. (Reprinted from Ref. [106]). Colors distinguish sensing, treatment, substrate, electrode, insulation, and communication modules and do not encode quantitative values.

Multiparameter systems require extra material organization because multiple readouts often share the same wound-contact region, exudate source, power or communication module, and, in some cases, a therapeutic function. Biomimetic microfluidic wound trackers extend this design logic to dynamic oxygen monitoring [105]. Stretchable bioelectronic systems further show how sensing, antimicrobial therapy, electrical stimulation, and soft electronic components can be integrated at the wound interface [106] (Figure 14), providing a current example of multimodule integration in wound-contact platforms. These systems are especially relevant to a materials-focused review when they document the physical separation of readout zones, the routing of exudate among modules, and the protection of sensing interfaces from adjacent therapeutic functions.

Crosstalk in multimodal dressings begins at the material and fluid levels before the signal reaches the data-processing stage [107]. Sensors drawing from the same wound-exudate pool may receive samples with different residence times. Adjacent optical and electrochemical windows may also interact, while heating, drug release, or electrical stimulation may change the monitored variables. Deformation introduces another source of error by changing contact area and channel geometry. Image-processing algorithms standardize data acquisition, although they do not replace material-level control of optical background, sample exposure, and interfacial stability. Wireless communication and data processing can improve usability, but they cannot compensate for inadequate readout separation, uncontrolled sample routing, or instability at the sensing zone.

#### 2.4.5. Readout-Dependent Evidence Map

Table 3 separates analytical performance from biological relevance for representative systems. Range, limit of detection (LOD), response time, interference testing, repeated-use evidence, test medium, and biological validation are reported independently. NR is used when the cited study did not provide a directly comparable value. This format prevents a low LOD in buffer from being interpreted as evidence of reliable wound monitoring.

The comparison in Table 3 changes the interpretation of apparently favorable results. The BC-MSN-BTB system responds within seconds in buffer and within 1 min on a porcine wound, whereas its 14-day stability endpoint is color retention in water [31]. The uric-acid OECT has a 702 s response and reports eight-day same-device reproducibility under simulated-exudate flow [98]. The MMP-9 platform uses a 24 h concentration-dependent response and reports agreement with an independent fluorescence assay in patient exudate, but the sample number is not stated [101]. The DNA-hydrogel system also requires 24 h for classification and reports 48 h wireless tracking [102]. These endpoints represent different transport distances, recognition reactions, decision purposes, and failure modes; the table supports mechanism-specific comparison, not a single ranking by LOD or response time.

## 3. Wet-State Reliability, Validation, and Translational Design Challenges

### 3.1. Wet-State Failure Modes and Signal Drift

A hydrated wound dressing is part of the sensing environment instead of an inert support, so changes in the material can directly alter readout accuracy. Under wound-like conditions, water uptake, salts, proteins, cellular debris, bacterial residues, and repeated deformation affect polymer domains that also govern wound contact, fluid routing, and signal transduction [108]. Swelling modifies diffusion and analyte exposure; loss of indicators or enzymes reduces signal-generating capacity; fouling limits access to the sensing surface; instability of conductive networks disrupts electrical pathways; and deformation changes channel geometry or electrode contact.

Analytical failure can often be traced to a specific physical change in the dressing. Swelling, for example, may lengthen diffusion pathways or alter the optical path through a colorimetric region, whereas dye loss can decouple observed color from local pH. Protein adsorption may shift the electrochemical baseline or mask recognition sites [109]; and bending or compression can alter reservoir volume, capillary filling, channel continuity, or electrode contact. These physically distinct processes can nevertheless produce similar experimental signatures, including drift, attenuation, delayed response, or incomplete recovery.

#### 3.1.1. Hydration, Swelling, and Diffusion

**Figure 15:**
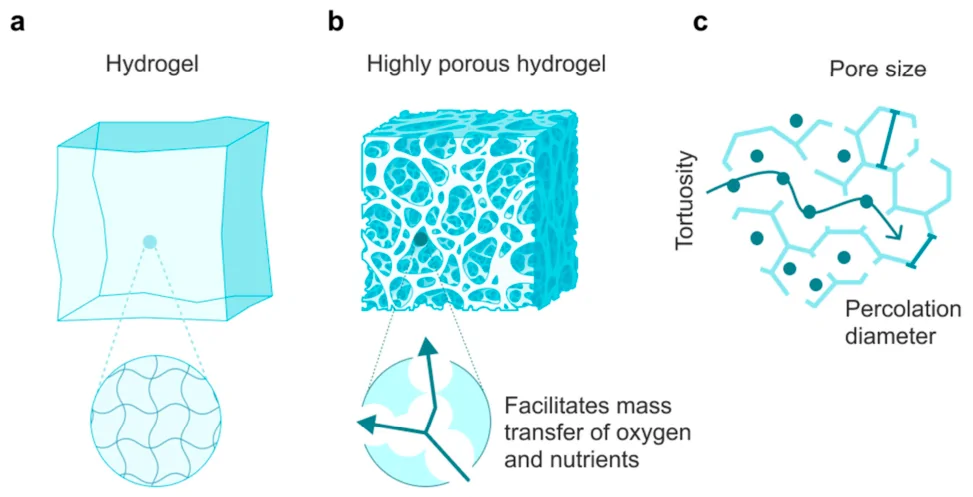
Hydrogel-network determinants of molecular transport. (**a**) A hydrated polymer network forms the base hydrogel matrix. (**b**) A highly porous network opens pathways that facilitate molecular transport. (**c**) Pore size, tortuosity, and percolation diameter govern diffusion and therefore the time- and concentration-dependent presentation of analytes to embedded sensing domains. (Reprinted from Ref. [110]).

Hydration is essential for wound contact, but it also reshapes the sensing environment. Hydrogel, cellulose, sponge, and electrospun matrices differ in exudate uptake and retention, while pore size, tortuosity, network density, and viscosity alter fluid penetration and residence time [110]. The diffusion scaling tD approximately L^2/Deff predicts that swelling-induced increases in path length can delay response even when equilibrium sensitivity is unchanged. Wet-state failure should be evaluated by paired endpoints: mass or volume uptake, thickness change, response and recovery time, signal attenuation, wet-dry hysteresis, reservoir-volume change, and maintenance of sample-sensor contact. Such measurements explain why dressings using the same dye, electrode, or enzyme can report different wound states after different upstream transport histories. These hydrogel-network determinants of molecular transport are summarized in Figure 15. Reversible pH 4/7 cycling (*n* = 2); color retained 14 days in waterPBS and SWE under flow; ISO 10993-5 [111] cytotoxicity; no real exudate/in vivo testMouse wounds *n* = 2/group; patient-derived test: 3 S. aureus-positive and 5 low-colonization samplesMouse *n* = 2/group; patient-derived assay 3 positive and 5 low-colonization samplesIV/*n* = 2 per group and no human performance study.

#### 3.1.2. Functional-Phase Retention and Matrix Interference

**Figure 16:**
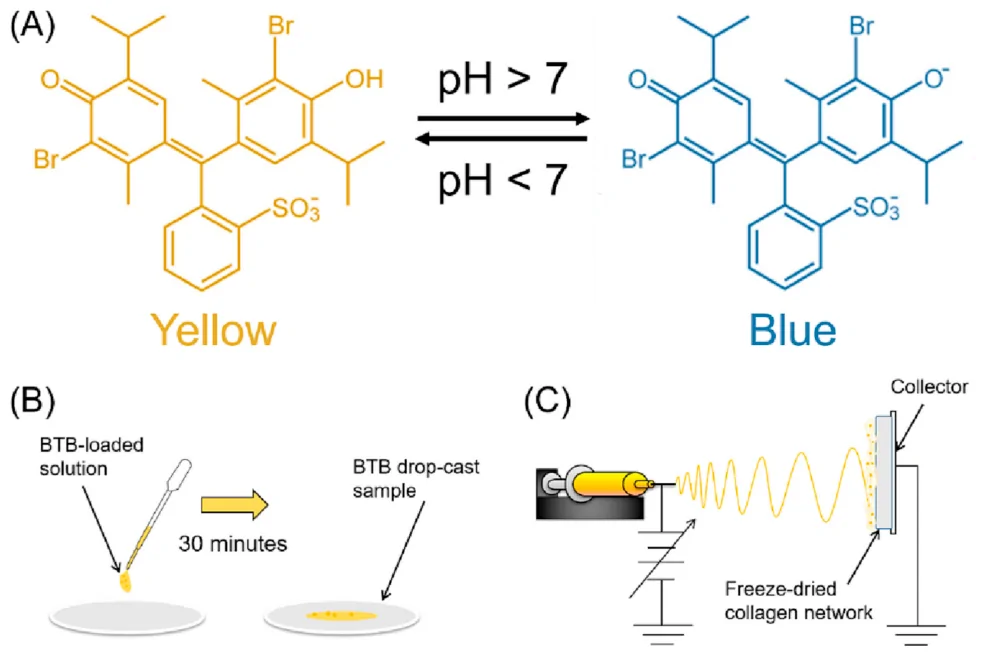
Retention and placement of bromothymol blue within a collagen-based wound dressing. (**A**) Bromothymol blue undergoes a reversible yellow-to-blue transition across the near-neutral pH range. (**B**) Drop-casting localizes the indicator on a collagen substrate. (**C**) Electrospinning deposits a dye-loaded sensing layer onto a freeze-dried collagen network. (Reprinted from Ref. [112]).

Functional phases must remain retained within the matrix and accessible to the target analyte. A collagen-based wound dressing illustrates this requirement by placing bromothymol blue in drop-cast and electrospun sensing layers above a collagen contact domain [112] (Figure 16). Nanocellulose protease-sensing dressings extend the same positional principle to enzymatic and protease-responsive readouts. Enzyme coatings and antibody-based interfaces likewise require both activity retention and recognition-site accessibility in protein-rich media. Evaluation should determine whether the dye, enzyme, or antibody remains localized, active, and accessible after soaking, repeated measurements, and wound-fluid exposure.

Proteins and other exudate components may alter the apparent response, redistribute fluid, or increase nonspecific adsorption [109]. In fibrous and paper-based devices, deposited material may narrow or block channels. Bacterial residues and biofilm fragments add further variability at the interface. Initial calibration in clean buffer does not reveal these effects. More informative studies combine retention measurements with recovery tests, deliberate fouling challenges, and post-use inspection. This combination helps distinguish loss of functional activity from restricted sample access.

#### 3.1.3. Conductive Phases and Mechanical Use Conditions

Electronic and transistor-based readouts introduce failure modes specific to hydrated conductive networks. Swelling and deformation may alter ion transport, disrupt contact at the channel interface, or break continuity along a conductive pathway. Flexible polypyrrole pH sensors demonstrate that a conductive interface remains functional under hydrated conditions [113]. Their relevance to wound monitoring, however, is determined by whether the baseline, electrode connections, and deformation response remain stable throughout the intended period of use.

A dressing experiences mechanical disturbance throughout use. It bends with the body and is exposed to compression, friction, and changes in humidity before eventual removal from the adhesive interface. These events may collapse fibrous networks, alter hydrogel volume, delaminate layers, foul the sensing window, or damage conductive tracks. Device-level studies of e-ECM platforms and holographic hydrogel bandages have examined the combined effects of soft substrates, wound-fluid exposure, and optical or electrical readout geometry [114,115]. The broader soft-bioelectronics literature likewise identifies body movement and physiological activity as persistent sources of signal artifacts [116].

#### 3.1.4. Reporting Priorities for Wet-State Failure Analysis

**Figure 17:**
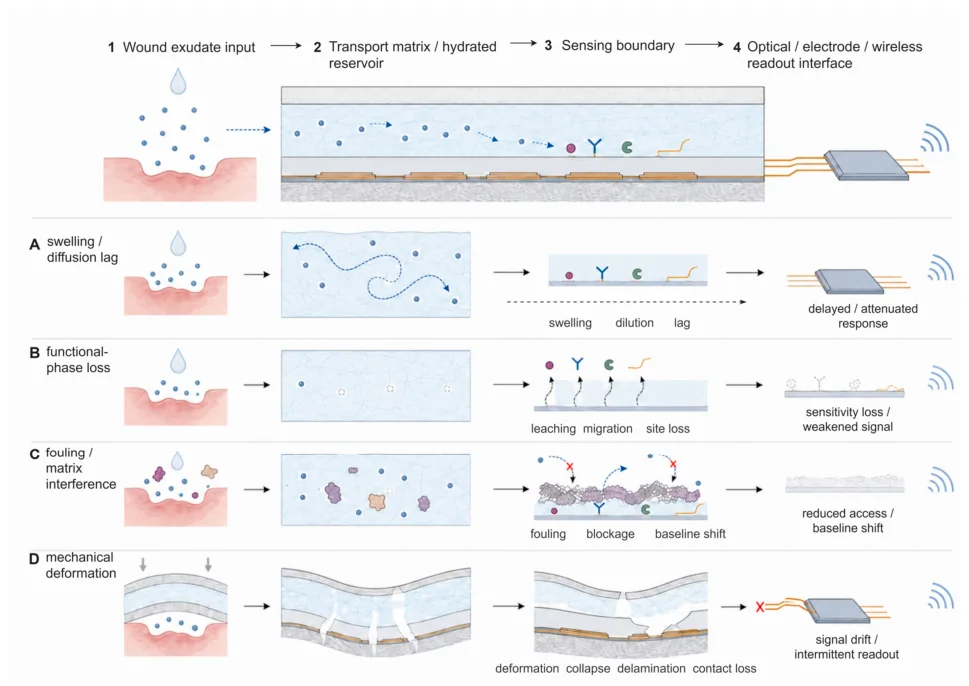
Wet-state failure pathways in sensor-integrated biobased polymer composite dressings. Wound exudate moves through a hydrated transport matrix to a sensing boundary and optical, electrode, or wireless readout interface. (**A**) Matrix swelling and sample dilution increase diffusion distance and response lag. (**B**) Leaching, migration, or functional-phase loss reduces sensitivity. (**C**) Fouling, blockage, and matrix interference restrict analyte access and shift the baseline. (**D**) Mechanical deformation, collapse, delamination, or contact loss disrupts interfacial or conductive continuity. Horizontal arrows trace the pathway from wound input through the transport matrix and sensing boundary to the external readout; colored markers represent transported sample components rather than quantitative values.

Failure modes can be assigned to the sampling, active-phase, interface, and device levels. Swelling, dilution, dead volume, or diffusion lag may change the sample before it reaches the sensor. Within the active phase, dye leakage, enzyme loss, nanoparticle migration, or conductive-network drift may weaken signal generation. Fouling, channel blockage, and matrix-dependent calibration shifts occur at interfaces between these stages. Deformation, wet aging, performance loss during use, and batch variability arise at the device and use levels. The relevant failure mechanism is the material change that converts a wound-derived input into a delayed, attenuated, unstable, or incorrect output [117], as illustrated in Figure 17.

The most informative acceptance endpoint depends on the readout mechanism. Colorimetric dressings should report spectral or calibrated color change together with retained dye percentage, leachate signal, response/recovery time, and performance after repeated pH cycles. Enzyme- and antibody-based sensors require retained catalytic or binding activity, recovery of spiked analyte in complex fluid, and baseline drift after soaking. Conductive dressings require sensitivity retention, resistance or baseline change after specified bending/stretching cycles, and interdevice variability. Microfluidic systems should quantify filling time, dead volume, leakage, backflow, incomplete filling, and signal recovery across defined viscosities and compressive loads. Rather than imposing a universal numeric pass/fail value, studies should predefine an application-specific acceptance window and report the percentage change from the pre-exposure calibration [118].

A minimum wet-state reliability panel should include: (i) calibration before and after prolonged exposure to simulated exudate; (ii) response and recovery after repeated concentration or pH steps; (iii) mass loss and dye, enzyme, nanoparticle, or ion release; (iv) wet mechanical strength or modulus retention; (v) electrical continuity after cyclic deformation; and (vi) post-use inspection of layers, channels, adhesive boundaries, and sensing windows. Reporting test temperature, fluid composition, volume, renewal rate, mechanical load, cycle number, and replicate number is necessary for reproducibility. These endpoints convert qualitative claims of stability into measurable evidence and reveal whether failure arises in sampling, signal generation, the interface, or the assembled device.

### 3.2. Validation Framework and Translational Evidence

Validation is organized here into five levels. Level I is calibration in buffer or a single defined solution. Level II adds simulated wound fluid, deliberate interferents, and, where relevant, controlled flow or routing. Level III uses complex real or ex vivo wound exudate and an independent reference assay. Level IV evaluates the integrated dressing in an animal wound model under use-relevant attachment, deformation, and fluid exposure. Level V comprises prospective human wound-sample or clinical-use validation against a defined reference method and decision threshold. The levels identify the highest context demonstrated; they are not substitutes for one another, because an animal study without analytical recovery or agreement testing does not repair a weak Level II calibration. Claims should remain proportional to the evidence available at each level [119].

#### 3.2.1. Evidence Levels of Current Platforms

Table 4 makes the framework operational by linking each level to a minimum test context, required evidence, comparator, and claim ceiling. Advancement is cumulative in interpretation but not automatic in evidence: a Level IV animal demonstration still requires Level I calibration and Level II matrix controls, and it does not support a clinical claim without Level V evaluation. Analytical range and LOD characterize the transducer, whereas matrix recovery, interference, reference-method agreement, sample size, and failed-sample reporting determine whether the result is biologically interpretable. Table 5 applies this distinction to representative systems by separating sensing outcomes from therapeutic outcomes and recording the highest context demonstrated [120,121,122,123,124,125,126].

Table 5 uses NR where information was not reported or could not be compared directly. The absence of a Level V system in this representative set is consequential: most platforms demonstrate function in controlled or preclinical settings without prospective comparison against a clinical decision standard.

**Table 5:** Validation evidence and remaining gaps for representative sensor-integrated wound-dressing systems. Level I, defined solution; Level II, simulated wound fluid/interference or controlled routing; Level III, real or ex vivo wound exudate with reference comparison; Level IV, integrated animal wound model; Level V, prospective human/clinical validation. NR, not reported.

Study/Platform	Reference Assay	Agreement or Analytical Evidence	Sample Size/Model	Sensing Outcome	Therapeutic or Biological Outcome	Highest Level/Principal Remaining Gap	Ref.
Arcangeli et al.; textile OECT for uric acid	Prepared UA standards; no clinical comparator	100–1000 micromolar in SWE; LOD 23 micromolar; t90 702 s	14 devices for reproducibility; no animal/human samples	Reversible UA monitoring under SWE flow	Cytotoxicity only; no therapeutic outcome	II/no real exudate or clinical comparator	[98]
Deng et al.; wireless MMP-9 dressing	Fluorescence assay	R2 0.9429; slope about 1; mean difference 0.20 microgram/mL; 95% of differences within 2 microgram/mL	DFU exudate sample number NR	MMP-9 measured in patient exudate	Controlled AgNP release and antibacterial testing	III/sample number and prospective threshold NR	[101]
Xiong et al.; DNA-hydrogel WINDOW	Quantitative wound culture/CFU	Detectable at 10^5^ CFU after 24 h; patient-derived samples classified by culture	Mouse n = 2/group; patient-derived assay 3 positive and 5 low-colonization samples	Wireless infection alert before visible signs	No treatment module	IV/n = 2 per group and no human performance study	[102]
Pang et al.; temperature sensor plus UV-responsive hydrogel	Environmental chamber; electronic thermometer control; Gram/HE staining	38–42 degrees C calibration; 15 min stability tests; formal method agreement NR	Bama minipig infected wounds; two wounds/group at each harvest; animal count NR	Continuous wound-temperature monitoring	Triggered gentamicin release; bacterial and inflammatory histology outcomes	IV/animal number and formal sensor agreement NR	[120]
Zhang et al.; GelMA/beta-CD sensor dressing	Constant-temperature chamber; paired gauze wound control	Mostly <0.3 degrees C deviation; <30 s response; no significant deviation change through 5 days in medium	Five rabbits with bilateral paired wounds	Continuous wound-temperature trend	Angiogenesis, inflammation, and wound closure	IV/clinical threshold and human validation absent	[121]
Ge et al.; all-hydrogel integrated dressing	Reference temperature/strain methods NR	Temperature and strain response reported; formal agreement NR	Rat wound model; n NR	Temperature and strain monitoring	Photothermal/on-demand drug delivery, antibacterial activity, and healing	IV/reference methods and animal n NR	[122]
Ge et al.; wireless closed-loop exudate-management dressing	Reference temperature/humidity methods NR	Sensor characterization reported; formal agreement NR	Mouse infected-wound model; n NR	Temperature/humidity monitoring with smartphone control	On-demand release; inflammation, angiogenesis, and collagen outcomes	IV/reference methods and animal n NR	[123]
Zong, Deng, and Huang systems; pH-responsive therapeutic hydrogels	Prepared pH standards; histology and inflammatory markers; independent wound-pH comparator NR	Visual/NIR pH response reported; formal clinical agreement NR	Diabetic or infected animal wound models; n NR in the comparative reports	Real-time or visual pH indication	Antibacterial/antioxidant, ultrasound-triggered delivery, and wound-healing outcomes	IV/independent wound-pH comparator and n NR	[124,125,126]

Contextual progression in Table 5 is not matched by analytical completeness. The MMP-9 dressing reaches Level III and provides patient-exudate comparison with a fluorescence assay, but its sample number and prospective decision threshold are not reported [101]. Six of the eight representative entries reach Level IV because an integrated animal model was used; among them, four do not report a complete animal sample size, and four lack a formal reference-method agreement analysis [102,120,121,122,123,124,125,126]. These examples show why model complexity and validation rigor must be reported as separate dimensions.

#### 3.2.2. Matrix-Aware and Dressing-Level Validation

**Figure 18:**
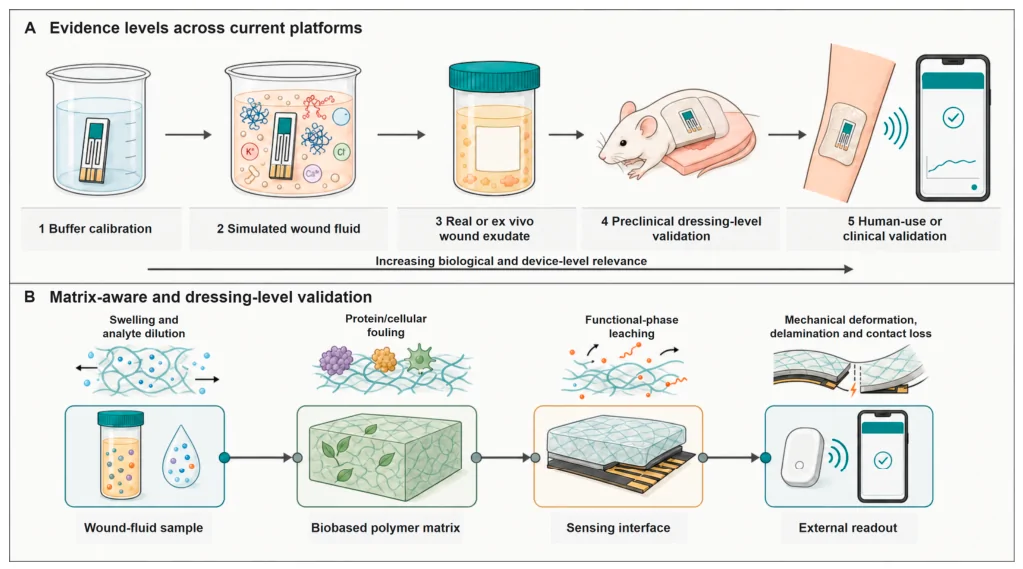
Five-level, matrix-aware validation framework for sensor-integrated biobased polymer wound dressings. (**A**) Evidence progresses from Level I buffer calibration and Level II simulated wound fluid/interference testing to Level III real or ex vivo exudate with reference comparison, Level IV integrated animal wound testing, and Level V prospective human or clinical validation. (**B**) At dressing level, the wound-fluid sample, polymer matrix, sensing interface, and external readout form a coupled pathway perturbed by swelling and dilution, protein or cellular fouling, functional-phase leaching, and mechanical deformation. Arrows indicate increasing biological and device-level relevance from Level I to Level V; colors distinguish material, interface, and failure-mode categories and do not encode quantitative values.

Matrix-aware validation asks whether the polymer composite changes access to the sensing surface before or during measurement. A potentiometric chip coated with a low-swelling zwitterionic hydrogel provides a direct example: protein, bacterial, and cellular adsorption were compared with an uncoated sensor, and the antifouling interface was then evaluated during on-body wound-pH monitoring [127]. Dressing-level validation extends the analysis to swelling, moisture-vapor transmission, retention under load, leakage, compression-induced release, wet mechanics, and post-use inspection. Optical regions should be checked for staining or dye loss, electrochemical systems for baseline and sensitivity recovery, and microfluidic devices for leakage, deformation, or incomplete filling. Figure 18 places these measurements within the five-level framework.

### 3.3. Translational Bottlenecks: Crosstalk, Manufacturing, Sterilization, and Storage

Theranostic and multimodal platforms introduce an additional validation layer because therapeutic interventions may alter the same variables being monitored. The pH-responsive, photothermal, and conducting-polymer systems discussed above indicate how drug release, localized heating, pH sensing, uric acid detection, and electrically triggered treatment may occur within a shared microenvironment. For the integrated systems summarized in Table 3, comparisons among sensor-only, treatment-only, blank-material, and fully integrated configurations are essential for distinguishing genuine biological changes from treatment-induced signal distortion. Controls must distinguish changes in wound state from signal shifts caused directly by the treatment.

**Figure 19:**
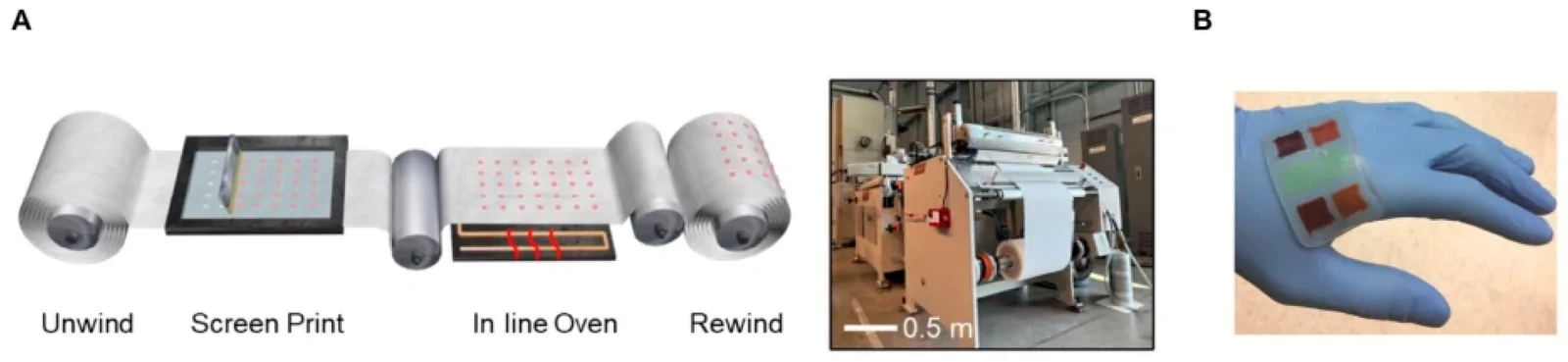
Manufacturing and use considerations for smart wound dressings. (**A**) Roll-to-roll screen printing with inline drying enables continuous and scalable production of sensor-patterned dressing substrates. (Adapted from Ref. [129]). (**B**) A multifunctional hydrogel dressing conforms to a curved surface while maintaining spatially separated sensing and drug-release domains. (Adapted with permission from Ref. [131]. Copyright 2017 WILEY-VCH Verlag GmbH & Co. KGaA, Weinheim).

The transition to manufacturable dressings introduces constraints that are largely absent from laboratory prototypes. In 3D-printed hydrogels, geometry and spatial deposition serve as controlled design variables [128]. Roll-to-roll screen printing with inline drying addresses a different set of requirements by enabling continuous production of patterned sensor substrates [129] (Figure 19A). Shelf-life studies further indicate that hydrogel dressings may undergo physicochemical changes during storage [130]. Multifunctional hydrogels with spatially separated sensing and drug-release regions provide a complementary use case because the functional domains remain distinct as the dressing conforms to a curved surface [131] (Figure 19B).

Sterilization and storage affect the complete sensing system, not only the hydrogel matrix. Radiation, ethylene oxide, heat, or disinfectant exposure may change polymer molecular weight, crosslink density, hydration, adhesion, channel geometry, dye spectra, enzyme or antibody activity, conductive-network resistance, electrode reference potentials, encapsulation, and wireless-module function [132,133]. Storage adds dehydration, oxidation, plasticizer migration, battery aging, adhesive change, and pre-use calibration drift. Paired pre/post-process tests should repeat the analytical calibration, wet-state transport test, mechanical/deformation test, functional-phase retention assay, and wireless readout check on the packaged final device. Shelf life should be assigned from the earliest component or performance endpoint that exits its predefined acceptance window.

Biological safety also has to be assessed for the assembled product and its released species. A risk-based program should address cytotoxicity, sensitization, irritation, extractables and leachables, and chemical characterization under ISO 10993 principles [111,134,135,136,137]. Residual crosslinkers, dyes, metal ions or nanoparticles, MXene oxidation products, conductive-polymer dopants, photothermal agents, adhesives, and degradation products require exposure-based justification instead of a generic claim of biocompatibility. Sterilization validation and packaging integrity should be aligned with the chosen process and terminally sterilized-device requirements [138,139]. Batch-to-batch polymer variation, printed-feature tolerances, calibration transfer, and combination of dressing, diagnostic, wireless, and therapeutic functions may place different modules under different regulatory controls; design history and acceptance criteria should be established at the integrated-product level.

The most consequential evidence gaps occur between calibration and use. Few reports combine matrix-matched recovery, independent method agreement, justified sample size, repeated wet-state testing, sterilized/aged-device performance, and prospective human evaluation. Practical minimum benchmarks are: recovery and interference testing across the intended wound-fluid range; response and recovery within the clinical sampling interval; predefined limits for baseline or sensitivity drift after wet aging and deformation; intact routing without leakage or backflow; retained functional phase and acceptable leachables; and agreement with a reference method reported with bias and limits of agreement, not correlation alone. Proof-of-concept studies remain valuable when they resolve a defined mechanism, but their claims should stop at the highest demonstrated validation level.

### 3.4. Design Considerations and Future Directions

Across the reviewed studies, material optimization and sensor calibration are often treated as separate tasks. This separation becomes consequential when calibration assumes that sample delivery is independent of the surrounding matrix. Tracing the sample through exudate uptake, transport, sensing, and signal interpretation makes the resulting gap visible by identifying the relevant fluid state, material-domain function, sensing-boundary stability, and supporting evidence at each stage.

Readout interpretation depends on the fluid state presented to the sensor. Freshly renewed exudate, reservoir-stored fluid, filtered samples, and direct tissue-boundary sampling are not analytically equivalent because polymer swelling, pore geometry, moisture-vapor transmission, and exudate viscosity can alter residence time and reshape concentration profiles before detection. Reporting the sampled fluid state together with material architecture would make platform-to-platform differences easier to interpret.

Material domains also require clear functional assignments. The wound-contact matrix, absorbent reservoir, capillary pathway, sensing host, protective coating, and electronic support layer face different requirements for surface charge, swelling, degradation, and wet-state mechanics. Multilayer and printed devices enable spatial separation of these requirements. A single-hydrogel design does not provide this separation and requires direct evidence that fluid storage, recognition, contact, and readout remain compatible within the same network.

Accessibility and retention define the sensing boundary. Dyes, enzymes, antibodies, conductive polymers, and nanoparticles remain useful only when they stay within the intended region and are accessible to the relevant fraction of wound fluid. Matrix-confined optical, enzymatic, and protease-responsive systems demonstrate this retention requirement. Electrical systems face a comparable risk when swelling or repeated use shifts a conductive network away from its calibrated state.

The validation sequence should be planned as an evidence chain. Buffer calibration establishes transducer response; standardized simulated wound fluid tests matrix effects; flow and replenishment experiments test routing; real or ex vivo exudate tests recovery and reference-method agreement; animal models test integrated attachment and operation; and prospective human studies establish clinical thresholds and workflow value. Storage, packaging, and sterilization must be inserted before the final analytical and dressing-level tests instead of being evaluated only on an isolated material. Closed-loop platforms add constraints on sensor position, communication, adhesion, treatment delivery, and biointerface stability, as illustrated by skin-interfaced bandages that integrate sensing, wireless communication, drug delivery, and dressing-level functions [140].

## 4. Conclusions

Biobased polymer composites influence wound sensing by controlling how exudate is contacted, transported, retained, and presented to the readout. Their value is architectural as well as biological. Molecular weight, substitution or deacetylation, polymer ratio, crosslink density, pore geometry, and functional-phase loading jointly determine swelling, wet mechanics, analyte partitioning, diffusion, retention, and signal stability. Reporting these variables alongside analytical performance is necessary for comparisons across chitosan, alginate, HA, cellulose, protein-derived matrices, and their conductive or responsive composites.

The principal bottleneck is not insufficient sensor sensitivity; it is the transfer of calibration from controlled media to a dynamic, fouling, deformable wound-dressing system. A material that responds in buffer may fail after reservoir dilution, adsorption, swelling, treatment-induced change, sterilization, storage, or mechanical use. Analytical range and LOD must therefore be interpreted separately from physiological relevance, reference-method agreement, and clinical decision value.

This narrative review has several limitations. The heterogeneity of materials, analytes, test media, device geometries, and outcome definitions precluded pooled comparison or meta-analysis. The English-language search and variable database indexing may have omitted relevant reports, while many included studies did not disclose composition, wet-state performance, sample size, reference-method agreement, or negative results in a comparable form. The proposed five-level framework is a practical evidence-mapping tool instead of a clinically validated grading scale. Quantitative values should be interpreted as study-specific unless media composition, conditioning, geometry, wound etiology, and outcome definitions are aligned.

A prioritized research roadmap follows from this gap: (1) standardize and fully report simulated wound-fluid composition and viscosity; (2) quantify exudate transport, partitioning, dead volume, and sample renewal; (3) validate antifouling, recovery, and selectivity in complex real or ex vivo fluid; (4) define acceptance limits for long-term wet-state signal and mechanical stability; (5) separate disease-induced changes from treatment-induced shifts using sensor-only, treatment-only, blank-material, and integrated controls; (6) verify calibration, biosafety, routing, electronics, and packaging after manufacturing, sterilization, and storage; and (7) conduct adequately powered prospective human validation against clinical reference methods and prespecified thresholds. Progress through these priorities would convert material responsiveness into reproducible, clinically interpretable wound monitoring.

## Figures and Tables

**Table 1:** Scope and analytical positioning of the present review relative to relevant review literature.

Review Category/Representative Refs.	Primary Organizing Unit	Polymer or Network Analysis	Exudate or Sample-Path Analysis	Sensing-Interface Reliability	Validation Emphasis
Broad wound materials and technologies [3]	Clinical application, material class and translation	Broad design principles across wound-care materials	Fluid management considered as a dressing function	Wearable biosensors included within a wider technology landscape	Bench-to-bedside translation and regulatory pathways
Smart and sensor-integrated dressing reviews [4,8]	Monitored parameters, sensing or responsive modality, and therapeutic function	Polymer classes identified as hosts for sensing, drug release, electroactivity, or wound contact	Moisture and exudate treated mainly as monitored variables or triggers	Sensor and treatment modalities mapped across responsive dressing designs	Preclinical evidence, clinical potential, and translational challenges
Wearable body-fluid analysis [5]	Body fluid, analyte, and wearable sensing modality	Not specific to biobased wound-dressing matrices	Sampling compared across multiple accessible body fluids	Emphasis on wearable analytical systems and clinical translation	Device translation across body-fluid applications
Natural-polymer wound-dressing reviews [11,12]	Polymer family, material format and wound-healing function	Synthesis, modification, crosslinking, and therapeutic performance	Absorption and moisture balance treated as wound-care properties	Integrated sensing is not the principal unit of comparison	Manufacturing, sterility, and stability discussed at material/product level
PVA-hydrogel and conductive-biomaterial reviews [13,14]	Crosslinking route, conductive phase, morphology, and biomedical use	Mechanical, conductive, and processing variables emphasized	Wet-state conductivity and exudate handling discussed for selected formats	Diagnosis or real-time monitoring is one application among several	Predominantly materials and preclinical challenges
Present review	Material architecture to sample pathway to sensing interface to evidence level	Molecular weight, substitution, composition, crosslink density and functional-phase loading	Routing, partitioning, adsorption, residence time and sensing-zone exposure	Wet-state drift, fouling, leaching, therapy-sensor coupling and device integrity	Five operational levels with claim ceilings and matrix-aware benchmarks

**Table 2:** Quantitative examples linking material and network variables to wet-state and sensing performance in biobased polymer composite wound dressings. Values remain study-specific and are not direct rankings. NR, not reported in the cited study set.

Matrix/Representative Composition	Molecular or Network Variables	Water Handling and Wet Mechanics	Sensor/Quantitative Response	Stability Evidence	Principal Limitation	Ref.
Alginate reservoirs and SA-containing composite fibers	Molecular weight; M/G ratio; Ca^2+^ crosslink density; blends with CMC, OHA or PVA	SA/PVA/CUR fiber: maximum swelling 266%; rapid uptake in 0–30 min and equilibrium by 1440 min	Visible response across pH 4–8, with the strongest transition around pH 6–7; response time NR	Curcumin release continued to 48 h; matched dye-retention and sensing-drift data NR	Reservoir dilution and diffusion lag; crosslinking-performance trade-off	[19,20,21]
HA and HA/chitosan soft interfaces	HA molecular weight; degree of substitution; Schiff-base and ionic crosslinking; polymer ratio	HC-KG hydrogel remained conductive under water; wound-fluid uptake and transport NR	Conductivity about 0.0638 S/m; gauge factor 2.64 for strain sensing	Repeatable 0–20% tensile cycling and post-self-healing conductivity; wound-analyte stability NR	Property variation with molecular weight/substitution; transport may slow as network density rises	[22,23,24]
Cellulose, nanocellulose, and BC; BC-MSN-BTB composite	Fibril packing, porosity, oxidation, MSN loading and PEI capping	Hydrated fibrous network; G′ > G″; MSN addition increased retention while preserving near-native WVTR	Colorimetric pH 5.5–8.5; seconds in buffer and <1 min in an infected porcine wound	Reversible pH 4/7 cycling; color retained for 14 days in water	Protein adsorption, optical background, and single-animal in vivo demonstration	[25,26,27,28,29,30,31]
Gelatin, collagen, and silk-fibroin contact/transition domains	Molecular-weight distribution; functionalization; enzymatic crosslinking or reinforcement	pH-responsive collagen fibers: swelling 323 +/− 79 to 492 +/− 73%; wet UTS 12 +/− 3 to 15 +/− 7 MPa	Reversible color transition between pH 5 and 8; response time NR	Dye loading 99 +/− 3 wt%; up to 88 wt% retained after 72 h; about 30 wt% crosslinked mass retained after 24 h collagenase exposure	Dry and wet mechanical data are rarely paired with sensing stability	[32,33,34,35,36]
Chitosan and quaternized chitosan interfaces	Controlled libraries span 1 to >100 kDa and 60–98% deacetylation; charge pattern and blend ratio also vary	Water uptake and wet stability depend on blending and crosslinking	No standardized wound-sensing response across the cited set	Keratinocyte and macrophage responses vary with molecular weight, dose, deacetylation, and acetylation pattern	Cationic benefits may be offset by cytocompatibility, adsorption, and matrix effects	[37,38,39,40,41]
Auxiliary PVA, conductive phases, MXenes, metals, and covalently bound dyes	Polymer/additive ratio; dispersion; percolation; oxidation state; immobilization chemistry	Alginate/PAAm-MAPR: original swelling ratio 251.5%; modulus 0.39 to 0.61 MPa as Ca^2+^ increased from 0.01% to 0.1%	Covalently bound phenol red changed from yellow to orange/red across pH 5–9; response time NR	Three-hour drying reduced swelling to 63.1% and raised modulus to 155 MPa; re-swelling did not restore the original modulus	Oxidation, hydrolysis, network drift, release, and treatment-sensor interference	[42,43,44,45,46,47,48,49,50]

**Table 3:** Standardized analytical-performance summary for representative sensor-integrated wound-dressing systems. NR, not reported; SWE, simulated wound exudate; LOD, limit of detection.

Platform/Analyte	Principle and Matrix	Range/LOD	Response	Selectivity/Interference	Cycles or Stability	Medium and Biological evidence	Ref.
BC-MSN-BTB dressing/pH	Colorimetric BTB retained in PEI-capped MSNs within BC	pH 5.5–8.5/LOD NR	Seconds in buffer; <1 min on infected porcine wound	Optical ratio reduces loading variation; wound color background evaluated qualitatively	Reversible pH 4/7 cycling (n = 2); color retained 14 days in water	Buffers; cultured cells; one porcine infected/noninfected wound pair	[31]
Alginate hydrogel fibers/pH	Dye-loaded mesoporous particles in Ca^2+^-crosslinked alginate/glycerol fibers	pH 6–9/LOD NR	Stable smartphone image after 30 min; exact t90 NR	Fiber diameter and optical channel evaluated; complex-fluid interference NR	Dye leaching reduced by particle loading; duration NR	Buffer and pH-treated ex vivo pig skin; no wound exudate or in vivo study	[96]
Textile OECT/uric acid	Direct oxidation on PEDOT:PSS textile transistor	100–1000 micromolar in SWE/23 micromolar	t90 = 702 s in SWE flow	pH 7–9 sensitivity RSD 5%; PBS versus SWE slope not significantly different	Same device over 8 days: RSD 3%; 14 devices: RSD 13%	PBS and SWE under flow; ISO 10993-5 cytotoxicity; no real exudate/in vivo test	[98]
Paper-based patch/oxygen	Luminescence sensing integrated with catalytic oxygen delivery	5–26 ppm/LOD NR	Oxygen increased by about 5 ppm in 1 h; sensor response time NR	Concurrent generation and sensing evaluated; wound-fluid interferents NR	Operational duration and cycle count NR	Gel model and mouse wound study; no clinical reference assay	[100]
Wireless GelMA sensor/MMP-9	Protease-cleavable hydrogel coupled to LC resonant-frequency readout	10–1000 ng/mL at 24 h/LOD NR	Concentration-dependent response at 24 h	Selectivity tested; DFU exudate compared with fluorescence assay	Stability tested; comparable duration value NR	DFU exudate: R2 = 0.9429, mean difference 0.20 microgram/mL; sample number NR	[101]
DNA-hydrogel WINDOW/bacterial DNase	DNase-mediated hydrogel degradation changes wireless capacitance	Detectable at 10^5^ CFU; saturation above 10^7^ CFU/LOD NR	24 h	Pathogenic versus commensal/cell selectivity assessed; culture used as reference	Wireless response followed for 48 h; bending fluctuation within +/−0.01 V	Mouse wounds n = 2/group; patient-derived test: 3 S. aureus-positive and 5 low-colonization samples	[102]

**Table 4:** Operational validation framework and claim ceiling for sensor-integrated wound dressings.

Level	Minimum Test Context	Required Analytical and Dressing Evidence	Comparator and Sampling Expectation	Maximum Supported Claim
I: defined solution	Buffer or single-analyte solution covering the intended range	Calibration function, range, sensitivity, LOD where applicable, response/recovery, hysteresis, stated, and device-to-device variation	Prepared standards and blank controls	Analytical response under defined solution conditions
II: matrix and routing	Composition- and viscosity-defined simulated wound fluid with interferents; controlled flow when routing is claimed	Matrix recovery, selectivity, fouling, pre/post-soak calibration, filling time, dead volume, leakage/backflow, leaching, and deformation stability	Spiked matrix controls, blank dressing, and predefined acceptance window	Matrix-compatible sensing or routing under specified simulated conditions
III: real or ex vivo sample	Real wound exudate or ex vivo wound fluid spanning the intended biological range	Recovery in the real matrix, bias and limits of agreement, failed-sample rate, sample handling, and retained device integrity	Independent reference assay; reported sample number and selection criteria	Agreement in the tested real or ex vivo sample set
IV: integrated animal use	Complete dressing on a defined wound model under attachment, motion, and fluid exposure	Longitudinal sensor output, paired reference measurements, adhesion/routing integrity, post-use calibration, and separation of sensing from treatment effects	Prespecified animal and wound numbers, controls, and time points	Preclinical integrated-use feasibility, not clinical diagnostic performance
V: prospective human/clinical	Prospectively enrolled wounds representing the intended-use population and workflow	Prespecified endpoint and threshold, agreement or diagnostic performance with uncertainty, missing-data analysis, usability, and adverse-event reporting	Clinical reference method, justified sample size, and independent evaluation when feasible	Clinical performance only for the tested population, endpoint, and decision threshold

## Data Availability

No new data were created or analyzed in this study. Data sharing is not applicable to this article.
